# Treatment of Hypovitaminosis D With Cholecalciferol in Dogs With Protein‐Losing Enteropathies: A Randomized, Double‐Blind, Placebo‐Controlled, Clinical Trial

**DOI:** 10.1111/jvim.70147

**Published:** 2025-06-08

**Authors:** Sara A. Jablonski, Sarah B. Shropshire, Victoria E. Watson, Alison C. Manchester, Harry Cridge, Elizabeth M. Lennon, M. Katherine Tolbert

**Affiliations:** ^1^ Department of Small Animal Clinical Sciences College of Veterinary Medicine, Michigan State University East Lansing Michigan USA; ^2^ Department of Clinical Sciences College of Veterinary Medicine and Biomedical Sciences, Colorado State University Fort Collins Colorado USA; ^3^ Department of Pathobiology and Diagnostic Investigation College of Veterinary Medicine, Michigan State University East Lansing Michigan USA; ^4^ Department of Small Animal Clinical Science University of Pennsylvania, School of Veterinary Medicine Philadelphia Pennsylvania USA; ^5^ The Gastrointestinal Laboratory, Department of Small Animal Clinical Sciences College of Veterinary Medicine and Biomedical Sciences, Texas A&M University College Station Texas USA

**Keywords:** 25OHD, canine, cholecalciferol, protein‐losing enteropathy, vitamin D

## Abstract

**Background:**

The effects of vitamin D supplementation are unknown in dogs with protein‐losing enteropathy (PLE).

**Objective:**

To evaluate the safety, efficacy, and clinical benefit of orally administered cholecalciferol in dogs with PLE and decreased serum concentrations of 25OHD.

**Animals:**

Twenty‐eight dogs with PLE, decreased 25OHD, and serum ionized calcium (iCa) > 1.0 mmol/L (*n* = 15 treated with cholecalciferol, *n* = 13 treated with placebo).

**Methods:**

Prospective, double‐blinded, randomized, controlled trial. Dogs randomized to receive 400 IU/kg cholecalciferol or placebo PO daily along with standard therapy for 6 weeks. Clinical and biochemical variables were measured at baseline (T0) and monitored at 2 (T1), 4 (T2), and 6 (T3) weeks postmedication initiation. Clinical and biochemical variables were also measured 6 weeks following discontinuation of study medication (T4). Variables were compared in dogs with PLE receiving cholecalciferol versus placebo at T0–T4 using Student's *t* test or Mann–Whitney U tests and a mixed‐effects model. Correlations between 25OHD and clinical and biochemical variables were also performed.

**Results:**

Dogs with PLE treated with cholecalciferol had higher 25OHD concentrations at T2 compared to dogs treated with placebo (225 nmol/L, range 72–434 vs. 80 nmol/L, range 31–254 nmol/L; *p* = 0.004). Clinical and biochemical variables did not otherwise differ between dogs with PLE treated with cholecalciferol versus placebo at T0–T4. Serum albumin correlated with 25OHD at T0–T3(*p* < 0.005 for all comparisons). Hypervitaminosis D without ionized hypercalcemia occurred in five dogs (18%).

**Conclusions:**

While PLE dogs treated with cholecalciferol had higher 25OHD concentrations at study timepoints, a clinical benefit of supplementation was not observed.

**Abbreviations**
25OHD: 25‐hydroxyvitamin‐D
BCS: body condition score
CD: Crohn's disease
CIE: chronic inflammatory enteropathy
CLIA: chemiluminescent immunoassay
cPLI: canine pancreatic lipase immunoreactivity
CRP: C‐reactive protein
CSU: Colorado State University
EPI: exocrine pancreatic insufficiency
GI: gastrointestinal
IBD: inflammatory bowel disease
iCa: ionized calcium
IL: intestinal lymphangiectasia
iMg: ionized magnesium
MCS: muscle condition score
MSU: Michigan State University
PFS: Purina fecal score
PLE: protein‐losing enteropathy
PTH: parathyroid hormone
RIA: radioimmunoassay
T0: time of enrollment
T1: 2 weeks following study drug initiation
T2: 4 weeks following study drug initiation
T3: 6 weeks following study drug initiation and time of study drug discontinuation
T4: 6 weeks poststudy drug discontinuation
TLI: trypsin‐like immunoreactivity
UC: ulcerative colitis
VDBP: vitamin D binding protein
VMC: Veterinary Medical Center
VTH: Veterinary Teaching Hospital

## 1 Introduction

Protein‐losing enteropathy (PLE) is a syndrome of excessive protein loss across a diseased enteric mucosa [1, 2]. It occurs as a consequence of a wide variety of disorders; however, chronic inflammatory enteropathy (CIE) and intestinal lymphangiectasia (IL) are considered the most common causes in dogs [1]. Dogs with PLE syndrome have a variety of clinical and biochemical abnormalities directly related to intestinal protein loss or malabsorption [1, 3]. Among those abnormalities, concentrations of serum vitamin D below the reference interval are common and could occur with or without concurrent decreases in serum ionized calcium (iCa) and secondary hyperparathyroidism [4, 5, 6]. The pathogenesis of serum vitamin D concentrations below the reference interval in dogs with PLE is not completely understood but is likely multifactorial and related to malabsorption, systemic inflammation, and direct loss [6, 7]. Dogs with PLE and serum concentrations of vitamin D below the reference interval have a worse outcome when compared to dogs with PLE and normal serum concentrations of vitamin D [8].

Vitamin D deficiency occurs in children with IL and in children and adults with inflammatory bowel disease (IBD), such as Crohn's disease (CD) and ulcerative colitis (UC) [9, 10, 11, 12]. In a study of 120 children with IBD and hypovitaminosis D, children supplemented with cholecalciferol (D3) had decreased IBD activity scores, fecal calprotectin, and serum C‐reactive protein (CRP) when compared to a placebo group [13]. In another study, humans with IBD treated with vitamin D had decreased utilization of health care facilities over the 5‐year study time period, suggesting improved overall health and disease control [14]. Similar studies have not been performed in dogs.

Cholecalciferol is an inexpensive and widely available form of vitamin D that is effective at raising serum 25‐hydroxyvitamin‐D (25OHD) concentrations in dogs with atopic dermatitis without causing serious adverse effects [15]. However, the safety, efficacy, and possible clinical benefit of supplementation of cholecalciferol has not been evaluated in dogs with PLE.

The purpose of this prospective, double‐blinded, randomized, controlled clinical trial was to evaluate orally administered cholecalciferol in dogs with PLE and serum concentrations of 25OHD below the reference interval. Specifically, we aimed to evaluate whether cholecalciferol is effective at raising serum 25OHD concentrations without frequent incidence of iatrogenic hypervitaminosis D or ionized hypercalcemia, and whether dogs treated with cholecalciferol plus standard therapy have significant improvement in clinical or biochemical variables when compared to dogs treated with a placebo plus standard therapy.

## 2 Materials and Methods

### 2.1 Sample Size

A sample size calculation was performed using the primary endpoints of CCECAI, serum albumin, and serum 25OHD calculations. Data from previous studies were used to assist in the sample size calculation [6, 16]. For CCECAI, a conjectured difference of six between cholecalciferol (test) and placebo (control) populations was used, with a common standard deviation of 3.5. For serum albumin concentrations, a conjectured difference of 0.8 g/dL between populations was used, with a common standard deviation of 0.5. For serum 25OHD concentrations, a conjectured difference of 30 nmol/L between populations was used, with a common standard deviation of 20 nmol/L. For all endpoints, sample size calculation was performed for a two‐sided *t* test with alpha fixed at 0.05. With a desired power of 0.8, a sample size of *n* = 11, *n* = 12, and *n* = 10 per group was sufficient for CCECAI, serum albumin, and serum 25OHD, respectively. We elected to recruit 15 dogs per group (*n* = 30 dogs) to address the potential for dog dropout.

### 2.2 Dogs

Dogs presented to Michigan State University (MSU) Veterinary Medical Center (VMC) and Colorado State University (CSU) Veterinary Teaching Hospital (VTH) for evaluation of PLE syndrome were eligible for inclusion. To be eligible for inclusion, dogs had to have a history of signs of gastrointestinal disease (e.g., diarrhea, changes in appetite [polyphagia, hyporexia, anorexia], weight loss, vomiting) of at least 3 weeks duration, a canine chronic enteropathy clinical activity index (CCECAI) score [17] > 3, a serum albumin concentration of < 2.5 g/dL, and a serum concentration of 25OHD < 50 nmol/L. Exclusion of other relevant causes of hypoalbuminemia was required based on normal unfed or unfed and postprandial bile acid concentrations and negative urine dipstick protein or urine protein: creatinine ratio of < 0.5. Previous exclusion of intestinal parasitism (via fecal floatation, broad spectrum deworming, or both), hypoadrenocorticism (as evidenced by basal serum cortisol concentration > 2 μg/dL [55 nmol/L] [18] or normal response to ACTH), and focal gastric or intestinal neoplasia (as assessed by abdominal ultrasound with or without cytology or histopathology) as the cause of GI signs and hypoalbuminemia was required. Histologic evidence of inflammatory enteritis, IL, or both was also required. A single board‐certified veterinary pathologist reviewed all samples (VEW). Exclusion criteria included evidence of clinically relevant non‐GI illness (as assessed by routine hematology, serum biochemical profile, and abdominal ultrasound), serum concentration of 25OHD ≥ 50 nmol/L, or histologic evidence of intestinal neoplasia or infectious enteropathy. Additionally, dogs were not eligible for inclusion if they were consuming a diet for > 72 h in the preceding 3 weeks not formulated to meet Association of American Feed Control Officials vitamin D requirements (500 IU/kg DMB) or currently receiving or had recently received oral supplementation of any formulation of vitamin D, calcium, or glucocorticoids in the previous 14 days. Finally, because of the randomized placebo‐controlled nature of the trial, dogs were excluded if iCa concentrations were ≤ 1.0 mmol/L or clinical signs attributable to ionized hypocalcemia (muscle tremors, facial rubbing, seizure activity) were present.

### 2.3 Experimental Protocol

The experimental protocol was approved by the Institutional Care and Use Committee at MSU (IACUC ID # PROTO202200302) and CSU (IACUC #4009). The CCECAI score was determined at the time of enrollment and follow‐up timepoints through the use of an owner questionnaire. The appetite score was recorded separately (0 = normal appetite, 1 = mildly decreased appetite, 2 = moderately decreased appetite, 3 = markedly decreased appetite). Dogs meeting the above criteria were enrolled in the study after informed consent was obtained. The following information was recorded at enrollment (Timepoint 0/T0): age, sex, breed, body weight (kg), body condition score (BCS) [19], muscle condition score (MCS) [20], Purina fecal score (PFS) [21], duration of clinical signs (weeks), current diet, current medications, results of fecal testing, urinalysis, bile acid testing, abdominal ultrasound, and intestinal histopathologic evaluation. The type of inflammatory infiltrate (lymphoplasmacytic, lymphoplasmacytic with eosinophils, lymphoplasmacytic with neutrophils), the degree of inflammatory infiltrate (mild = 1, moderate = 2, marked = 3), and degree of lacteal and crypt dilation were recorded. If the lacteal occupied 0%–25% of the villus width, the degree was mild; 25%–50% was moderate; and 50%–75% was severe. Scores for the inflammatory infiltrate and lacteal dilation were based on the most severely affected villus in each case [22]. Other variables recorded at enrollment included serum concentrations of cobalamin, folate, canine pancreatic lipase immunoreactivity (cPLI), trypsin‐like immunoreactivity (TLI), 25OHD, iCa, ionized magnesium (iMg), parathyroid hormone (PTH), albumin, globulin, cholesterol, CRP [23] and vitamin‐D binding protein (VDBP) [6]. Following enrollment, dogs were randomized to receive cholecalciferol (Vitamin D3 [powder, Medisca, Data S1]) at a dose of 400 IU/kg/day (dose extrapolated from previous publication) [15] or placebo (Avicel microcrystalline cellulose; Data S2). Dogs were randomly assigned to treatment groups using the RAND function in Microsoft Excel. Medication was compounded by a specialty compounding pharmacy, and 42 days' worth of medication was mailed directly to the client. The medication was labeled with administration directions but not drug name, maintaining owner blinding. The number of days between T0 and starting the study medication was recorded. Cholecalciferol or placebo was planned to be administered in all dogs for a total of 42 days. Owners were required to maintain drug administration logs to ensure compliance (Data S3). Required follow‐up timepoints were at 2 (Timepoint 1/T1), 4 (Timepoint 2/T2), 6 (Timepoint 3/T3), and 12 (Timepoint 4/T4) weeks postinitiation of the study drug. Dogs were allowed to be treated with other therapies suitable for dogs with PLE including dietary modifications, glucocorticoids, cobalamin, antiemetics, and acid‐reducing medications as deemed necessary; however, additional supplementation with calcium or vitamin D‐containing products was not permitted, and an attempt was made to keep treatment protocols similar in all dogs. Removal from the study was required if serum 25OHD concentrations exceeded the upper limits of the reference interval at times prior to T3, or if ionized hypercalcemia occurred at any timepoint.

At each of the follow‐up timepoints (T1–T4), the following information was obtained: number of days since previous visit, current medications and specific form and dose of glucocorticoid in mg/kg/day, current diet, CCECAI score, weight (kg), BCS, MCS, PFS, serum albumin, globulin, cholesterol, 25OHD, iCa, iMg, PTH, CRP, and VDBP. After the T3 timepoint, study medication was discontinued, and the final study timepoint was T4 (6 weeks postdiscontinuation of study medication). Six‐to‐12‐month outcome was recorded where available as alive, dead due to PLE, or dead due to other cause.

### 2.4 Laboratory Assays

Measurements of serum concentrations of iCa and magnesium, 25‐hydroxyvitamin D (25OHD), and PTH were performed at the MSU Veterinary Diagnostic Laboratory using routinely performed and validated assays. All assays were performed on the same serum sample from each timepoint. The iCa and magnesium concentrations were determined using ion‐specific electrodes. Serum 25OHD concentrations were measured with a commercially available radioimmunoassay (RIA) kit that provides reagents necessary for extraction and quantitation of the analyte (Data S4) for the first 18 months of the study. Following this time, the RIA kit was discontinued, so further measurements with this kit were not possible, and the laboratory switched to a chemiluminescent immunometric assay (CLIA) for measurement of 25OHD. Thus, all samples performed after this time were measured with the CLIA assay (Data S5). Serum PTH concentrations were measured using a CLIA assay (Data S6). Serum VDBP concentrations were measured using a previously published [6] human‐specific ELISA, validated for measurement (Data S7) in the dog for research by the kit manufacturer (Immunogen, Waltham, MA).

### 2.5 Statistical Analysis

Data were assessed for normality. Descriptive statistics were calculated, and treatment and placebo groups were assessed for differences in age, body weight, sex, duration of clinical signs, and baseline (T0) CCECAI score, appetite score, PFS, and serum 25OHD, albumin, iCa, iMg, PTH, CRP, VDBP, cobalamin, and PLI concentrations with Student's *t* test, Mann–Whitney U tests, and Fisher's exact test. Additionally, the degree of inflammatory infiltrate, lacteal dilation, and crypt dilation were compared between groups using Mann–Whitney U tests. Student's *t* test or Mann–Whitney U tests were performed as indicated based on normality testing for comparison of # of days between timepoints, steroid dose, CCECAI score, appetite score, PFS, and serum 25OHD, albumin, iCa, iMg, PTH, CRP, and VDBP concentrations at all follow‐up timepoints (T1, T2, T3, T4) between the test and placebo groups. Additionally, the fold change in PFS, BCS, and serum albumin and CRP over all study timepoints were assessed using a mixed‐effects model followed by Šídák's or Tukey's multiple comparisons test as appropriate. Spearman (rank‐based) correlations were also performed to evaluate for a correlation between 25OHD and PFS, CCECAI, and serum albumin, cholesterol, iCa, iMg, PTH, VDBP, and CRP, and between serum albumin and VDBP. For Spearman testing, a statistically significant correlation score of (±) 0.3–0.5 was considered a weak correlation, (±) 0.5–0.7 a moderate correlation, and (±) 0.7–1.0 a strong correlation. Bland–Altman method comparison was also performed (*n* = 20) to look for agreement between the RIA and CLIA for 25OHD. All statistical analysis was performed with GraphPad Prism Version 9.4.1 (GraphPad Software Inc., La Jolla, California). *p* < 0.05 were considered significant.

## 3 Results

### 3.1 Dogs

**FIGURE 1:**
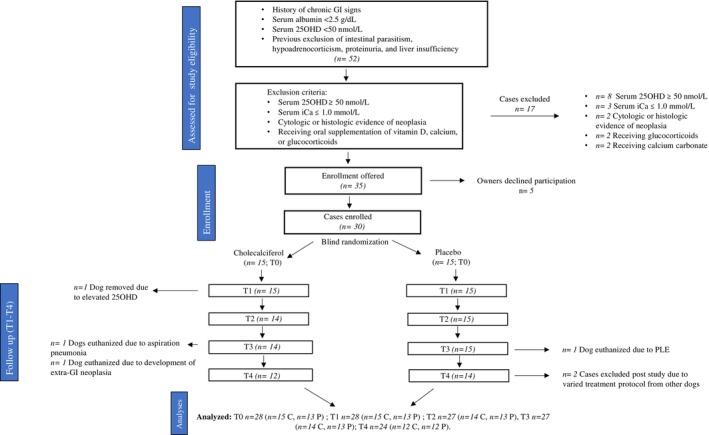
Flow diagram detailing case assessment, enrollment, follow‐up, and removal. C: cholecalciferol group; GI: gastrointestinal; *P*: placebo group; T1: 2 weeks postinitiation of study drug; T2: 4 weeks postinitiation of study drug; T3: 6 weeks postinitiation of study drug; T4: 6 weeks post‐T3 and discontinuation of study drug.

A total of 52 dogs were screened for inclusion in the study. Based on inclusion and exclusion criteria, 30 dogs were enrolled in the study with 28 dogs from MSU CVM and two dogs from CSU VTH. Upon completion of the study, it was discovered that all dogs except the two dogs from CSU were treated with glucocorticoids and both had been randomized to the placebo group. Therefore, to maintain consistency between the cholecalciferol and placebo populations, only the 28 dogs from MSU were included in the final data analysis. A flow diagram of case assessment, enrollment, and study progress is presented in Figure 1.

The median age of dogs in the cholecalciferol group was 7 years (range, 2–13 years). The median age of dogs in the placebo group was 8 years (range, 2–14 years). In the cholecalciferol group, seven dogs were spayed females, and eight dogs were castrated males. In the placebo group, seven dogs were spayed females, and six dogs were castrated males. The median weight of dogs in the cholecalciferol group was 27 kg (range, 3–56 kg); the median weight of dogs in the placebo group was 24 kg (range, 8–52 kg). Age, sex, and body weight were not different between groups. Breeds are shown in Table S1.

**FIGURE 2:**
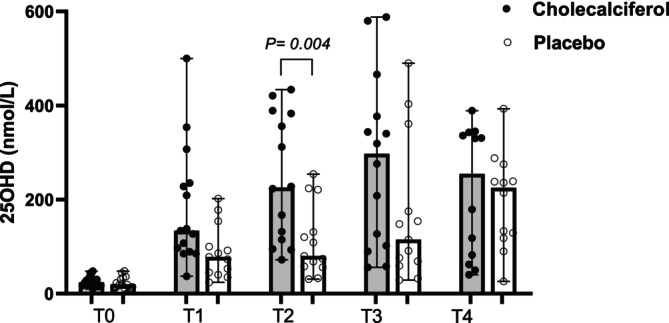
Scatter dot plot showing serum 25OHD concentrations in dogs with PLE receiving cholecalciferol versus placebo from T0 (baseline) to T4 (completion of study). Median and range shown. 25OHD: 25‐hydroxyvitamin‐D. *p* < 0.005 considered significant.

**FIGURE 3:**
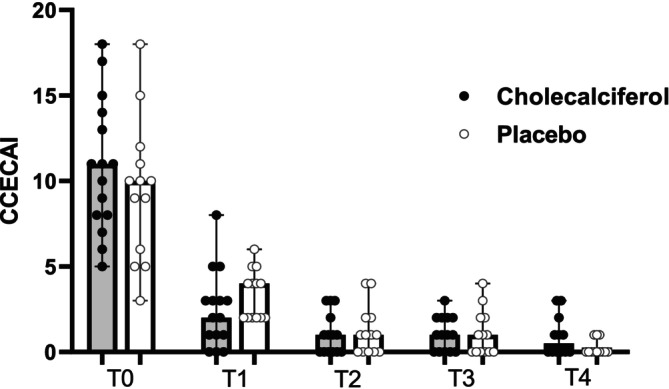
Scatter dot plot showing CCECAI scores in dogs with PLE receiving cholecalciferol versus placebo from T0 (baseline) to T4 (completion of study). Median and range shown. CCECAI: Canine chronic enteropathy activity index. All *p* > 0.05.

**FIGURE 4:**
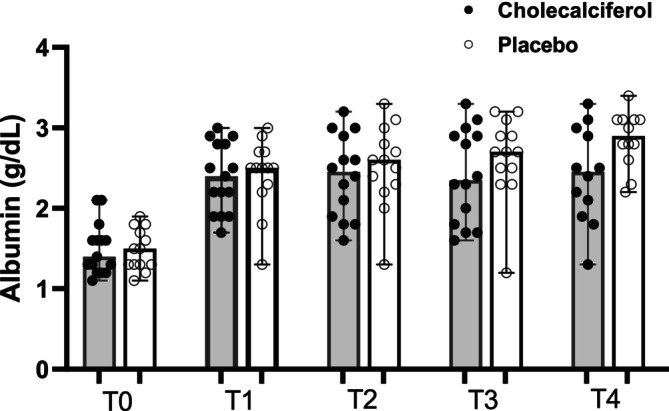
Scatter dot plot showing serum albumin concentrations in dogs with PLE receiving cholecalciferol versus placebo from T0 (baseline) to T4 (completion of study). Median and range shown. All *p* > 0.05.

The median duration of clinical signs in the cholecalciferol group was 8 weeks (range 3–30 weeks) compared to 10 weeks (range, 3–24) in the placebo group (*p = 0*.67). At T0 (enrollment) median CCECAI scores in the cholecalciferol and placebo groups were 11 (range 5–18) and 10 (range 4–18), respectively (*p = 0*.39). Appetite scores were also similar, with a median score of 0 and a range of 0–3 in both groups (*p = 0*.72). Median PFS at T0 was 6 for both groups, with a range of 4–7 for dogs randomized to the cholecalciferol group and a range of 3–7 for dogs in the placebo group (*p = 0*.44). Baseline (T0) biochemical data compared between groups is reported in Table 1. There were no significant differences in baseline biochemical data at T0 between groups (Table 1, Figures 2, 3, 4). Additional data describing abdominal ultrasound and histopathological findings are available in Table S2. The degree of inflammatory infiltrate (*p = 0*.79) and lacteal dilation (*p = 0*.17) were not different between groups.

**TABLE 1:** Baseline (T0) biochemical variables in dogs with PLE and decreased serum concentrations of 25OHD treated with cholecalciferol or placebo.

Variable	Reference interval	Cholecalciferol (*n* = 15) median (range) or Mean ± SD above^b^ or below^c^ RI	Placebo (*n* = 13) median (range) or mean ± SD above^b^ or below^c^ RI	*p* ^a^
25OHD (nmol/L)	109–423	26.1 ± 10.8	24.9 ± 12.4	0.78
		15/15 (100%)^c^	13/13 (100%)^c^	
Albumin (g/dL)	2.8–3.6	1.4 (1.1–2.1)	1.5 (1.1–1.9)	0.88
		15/15 (100%)^c^	13/13 (100%)^c^	
Cholesterol (mG/dL)	126–325	113 (81–179)	106 (53–202)	0.29
		10/15 (67%)^c^	10/13 (77%)^c^	
Ionized calcium (mmol/L)^d^	1.25–1.45	1.30 ± 0.09	1.31 ± 0.10	0.94
		3/15 (20%)^c^	3/15 (20%)^c^	
Ionized magnesium (mmol/L)	0.43–0.60	0.55 ± 0.15	0.51 ± 0.08	0.37
		4/15 (27%)^c^	4/13 (31%)^c^	
Parathyroid hormone (pmol/L)	1.10–10.6	9.6 (2.0–54.5)	8.0 (2.3–29.2)	0.61
		7/15 (47%)^b^	4/13 (31%)^b^	
Cobalamin (ng/L)^e^	251–908	238 (150–1000)	300 (150–532)	0.89
		8/15 (53%)^c^	6/13 (46%)^b^	
Folate (μg/L)	7.7–24.4	12.3 (6.9–18.8)	11.1	0.67
		2/15 (13%)^c^	2/13 (15%)^c^	
cPLI (μg/L)	≤ 200	113 (30–1334)	129 (30–637)	0.71
		4/15 (27%)^b^	4/13 (31%)^b^	
CRP (μg/mL)^f^	≤ 9.9	22.2 (6.9–146)	16.9 (6.9–134)	0.32
		12/16 (75%)^b^	9/13 (69%)^b^	
VDBP (μg/mL)	NA	29.8 (1.0–86.3)	32.8 (7.7–65.8)	0.97

Abbreviations: 25OHD: 25‐hydroxyvitamin‐D; CRP: C‐reactive protein; RI: reference interval; SD: standard deviation; VDBP: vitamin D binding protein.

^a^ *p* value as assessed by Mann–Whitney *U* for nonparametric variables (data presented as median range) and *t* test for parametric variables (data presented as mean ± SD).

^b^ Indicates proportion reported is number of dogs above the RI.

^c^ Indicates proportion reported is number of dogs below the RI.

^d^ Inclusion criteria required concentrations > 1.0 mmol/L.

^e^ Concentrations reported as < 150 were recorded as 150 ng/L.

^f^ Lower limit of detection for the assay is 6.9 μg/mL.

Following randomization into placebo or cholecalciferol group and discharge at T0, all dogs were transitioned to a prescription veterinary therapeutic diet or a homecooked diet formulated for their specific needs and supplemented with Balance It Canine (pure vitamin, mineral, and amino acid supplement). Diets prescribed in each group are shown in Data S8.

All dogs were prescribed prednisone or prednisolone at a median dose of 0.97 mg/kg, PO, q24h in the cholecalciferol group, and 1.0 mg/kg, PO, q24h in the placebo group (*p = 0*.17). Clopidogrel was prescribed for thromboprophylaxis for the duration of the study in all dogs in the placebo group and 14/15 (93%) dogs in the cholecalciferol group at a median dose of 2.5 mg/kg, PO, q24 in both groups. All dogs were receiving unflavored over‐the‐counter cobalamin supplementation at dosing appropriate for weight for the duration of the study. Infrequently prescribed medications are shown in Data S8. Three dogs in the placebo group and five dogs in the cholecalciferol group had their diet changed at least once during the study based on persistent diarrhea, hypoalbuminemia, or both. No dogs were treated with alternative forms of glucocorticoids or with secondary immunosuppressive agents.

### 3.2 Comparison of Variables at T1–T4

Clinical and biochemical data for the two groups at T1–T4 are reported in Table 2. The number of days between T0 and the initiation of the study drug and the number of days between T0 and T1 did not differ between groups (Table S3). At T1, the serum concentration of 25OHD was greater in the cholecalciferol group (134 nmol/L) versus the placebo group (78 nmol/L); however, this difference was not statistically significant after correcting for multiple comparisons (Figure 2). Steroid dose, CCECAI score (Figure 3), appetite score, PFS, and serum albumin (Figure 4), iCa, iMg, PTH, CRP, and VDBP concentrations did not differ between groups at T1. One dog at T1 had a serum concentration of 25OHD above the reference interval (500 nmol/L, RI, 109–423 nmol/L) and based on study protocol, the medication was discontinued, and the dog was removed from the study. Therefore, T2 and T3 contain *n* = 27 dogs (*n* = 13 placebo, *n* = 14 cholecalciferol). The number of days between T1 and T2, T2 and T3, and T3 and T4 also did not differ between groups. Two dogs (one each from the placebo and cholecalciferol dogs) were euthanized between T3 and T4 (see Outcomes). Additionally, one dog from the cholecalciferol group was diagnosed with extra‐gastrointestinal neoplasia at T4 and therefore excluded from the T4 analysis, leaving *n* = 24 (*n* = 12 placebo, *n* = 12 cholecalciferol) at T4. At T2 (*n* = 27), T3 (*n* = 27), and T4 (*n* = 24), the steroid dose, CCECAI score (Figure 3), appetite score, PFS, and serum albumin (Figure 4), iCa, iMg, PTH, CRP, and VDBP concentrations did not differ between groups. At T2, the serum concentration of 25OHD was again greater in the cholecalciferol group (225 nmol/L) versus the placebo group (80 nmol/L; *p = 0*.004; Figure 2). At T3 and T4, serum concentrations of 25OHD did not differ between groups (Figure 2).

**TABLE 2:** Clinical and biochemical variables at T1 (*n* = 28), T2 (*n* = 27), T3 (*n* = 27), and T4 (*n* = 24) in dogs with PLE and decreased concentrations of 25OHD treated with cholecalciferol or placebo.

Variable	Timepoint	Cholecalciferol (*n* = 15) median (range) or mean ± SD	Placebo (*n* = 13) median (range) or mean ± SD	*p* ^a^
CCECAI	T1	2 (0–8)	4 (2–6)	0.13
	T2	1 (0–3)	1 (0–4)	0.81
	T3	1 (0–3)	1 (0–4)	0.79
	T4	0.5 (0–3)	0 (0–1)	0.16
Prednisone/prednisone dose (mg/kg/day)	T1	0.97 ± 0.17	1.0 ± 0.27	0.57
	T2	0.75 ± 0.22	0.70 ± 0.23	0.91
	T3	0.54 ± 0.16	0.56 ± 0.17	0.74
	T4	0.34 ± 0.08	0.32 ± 0.17	0.62
25OHD (nmol/L); % within RI; % above RI	T1	134 (27–500); 46%; 6%	78 (24–202); 23%; 0%	0.01
	T2	225 (72–434); 79%; 0%	80 (31–254); 46%; 0%	**0.004**
	T3	297 (56–588); 64%; 21%	115 (29–490); 54%; 7%	0.15
	T4	254 (40–389); 75%; 0%	225 (26–393); 83%; 0%	0.77
Albumin (g/dL)	T1	2.4 (1.7–3.0)	2.7 (1.3–3.0)	0.71
	T2	2.5 (1.6–3.2)	2.6 (1.3–3.3)	0.49
	T3	2.4 (1.6–3.3)	2.7 (1.2–3.2)	0.35
	T4	2.5 (1.3–3.3)	2.9 (2.2–3.4)	0.06
Ionized calcium (mmol/L)^b^	T1	1.33 ± 0.04	1.31 ± 0.04	0.27
	T2	1.32 ± 0.05	1.35 ± 0.04	0.72
	T3	1.33 ± 0.04	1.34 ± 0.04	0.94
	T4	1.31 ± 0.05	1.35 ± 0.06	0.08
Ionized magnesium (mmol/L)	T1	0.60 ± 0.10	0.58 ± 0.09	0.60
	T2	0.61 ± 0.10	0.63 ± 0.07	0.38
	T3	0.60 ± 0.11	0.58 ± 0.09	0.58
	T4	0.59 ± 0.04	0.61 ± 0.08	0.29
Parathyroid hormone (pmol/L)	T1	9.4 (2.8–32.2)	10.5 (1.8–32.5)	0.77
	T2	7.5 (1.9–41.7)	7.9 (1.5–37.7)	0.88
	T3	7.9 (1.5–24.1)	8.0 (0.5–27.2)	0.78
	T4	7.5 (2.9–334)	4.5 (1.0–67)	0.14
CRP (μg/mL)^c^	T1	6.9 (6.9–29.12)	6.9 (6.9–6.9)	> 0.99
	T2	6.9 (6.9–36.7)	6.9 (6.9–6.9)	0.48
	T3	6.9 (6.9–34.1)	6.9 (6.9–17.2)	0.48
	T4	6.9 (6.9–65.6)	6.9 (6.9–6.9)	> 0.99
VDBP (μg/mL)	T1	31.1 (3.7–78.1)	30.1 (13.5–65.8)	0.59
	T2	33.8 (15.9–68.5)	32.1 (1.5–95.1)	0.54
	T3	29.1 (6.9–79.3)	27.7 (1.0–78.1)	0.80
	T4	30.7 (16.1–76.5)	26.1 (11.4–116.7)	> 0.99

*Note:* The bold values indicate significant *p* values after Bonferroni correction.

Abbreviations: 25OHD: 25‐hydroxyvitamin D; CCECAI: canine chronic enteropathy clinical activity index; CRP: C‐reactive protein; SD: standard deviation; VDBP: vitamin D binding protein.

^a^ *p* value as assessed by Mann–Whitney *U* for nonparametric variables (data presented as median range) and *t* test for parametric variables (data presented as mean ± SD). After Bonferroni correction, *p* < 0.005 was considered significant.

^b^ Inclusion criteria required concentrations > 1.0 mmol/L.

^c^ Lower limit of detection for the assay is 6.9 μg/mL.

### 3.3 Changes in Selected Variables Over Time

The fold change in PFS, BCS, and serum 25OHD, albumin, and CRP from baseline over the duration of the study was not different between the cholecalciferol and placebo groups (Table 3). The fold change in PFS, BCS, and serum 25OHD, albumin, and CRP from baseline differed depending on the timepoints being compared (Data S5).

**TABLE 3:** Mixed effects model examining the change in selected variables over time in dogs with PLE treated with either cholecalciferol or placebo.

Variable	Group^a^	Time^b^	Group × Time^c^
PFS	0.38	< 0.001	0.41
BCS	0.26	< 0.001	0.19
Albumin (g/dL)	0.53	< 0.001	0.60
CRP (μg/mL)^c^	0.69	< 0.001	0.85

Abbreviations: BCS: body condition score; CRP: C‐reactive protein; PFS: Purina fecal score.

^a^ *p* for a treatment‐dependent effect.

^b^ *p* for a time‐dependent effect.

^c^ *p* for a treatment‐by‐time effect.

### 3.4 Correlations

Serum 25OHD concentrations were positively correlated with serum albumin concentrations at all timepoints in the study. Additionally, serum 25OHD concentrations were positively correlated with serum cholesterol and negatively correlated with serum iCa, PTH, and CRP at T0. A negative correlation between serum concentrations of 25OHD and PFS was observed at T3. Additional correlation data is shown in Table 4. There was no correlation between serum albumin and serum VDBP at any time point (Table S3).

**TABLE 4:** Correlation between clinical and biochemical variables at T1 (*n* = 28), T2 (*n* = 27), T3 (*n* = 27), and T4 (*n* = 24) with serum 25OHD in dogs with PLE and concentrations of 25‐hydroxyvitamin‐D (25OHD) below the reference interval treated with cholecalciferol or placebo.

Variable	Timepoint	Spearman correlation score (rho)	*p* ^a^
PFS	T0	−0.2592	0.18
	T1	−0.2129	0.28
	T2	−0.3452	0.08
	T3	−0.4647	0.01
	T4	−0.2273	0.27
CCECAI	T0	−0.1339	0.49
	T1	−0.0743	0.71
	T2	−0.3951	0.04
	T3	−0.2365	0.23
	T4	−0.0935	0.66
Albumin (g/dL)	T0	0.5499	**0.002**
	T1	0.5210	**0.004**
	T2	0.5870	**0.001**
	T3	0.5506	**0.002**
	T4	0.4448	0.03
Cholesterol (mg/dL)	T0	0.4599	0.01
	T1	0.1664	0.49
	T2	0.4530	0.02
	T3	0.2720	0.17
	T4	0.2271	0.28
Ionized calcium (mmol/L)^b^	T0	0.5784	**0.001**
	T1	0.1048	0.59
	T2	0.1226	0.54
	T3	0.0320	0.87
	T4	0.1841	0.38
Ionized magnesium (mmol/L)	T0	0.1845	0.35
	T1	−0.1373	0.49
	T2	0.0412	0.84
	T3	0.0155	0.94
	T4	−0.3303	0.11
Parathyroid hormone (pmol/L)	T0	−0.6175	**< 0.001**
	T1	−0.2676	0.17
	T2	−0.4133	0.03
	T3	−0.2107	0.29
	T4	−0.3578	0.08
CRP (μg/mL)^c^	T0	−0.4575	0.01
	T1	−0.0251	0.90
	T2	−0.0162	0.84
	T3	−0.2187	0.28
	T4	−0.2832	0.17
VDBP (μg/mL)	T0	0.0896	0.65
	T1	0.0629	0.76
	T2	0.1493	0.46
	T3	0.0219	0.91
	T4	0.0080	0.97

*Note:* The bold values indicate significant *p* values after Bonferroni correction.

Abbreviations: 25OHD: 25‐hydroxyvitamin‐D; CCECAI: canine chronic enteropathy clinical activity index; CRP: C‐reactive protein; PFS: Purina fecal score; VDBP: vitamin D binding protein.

^a^ *p* as assessed by Spearman correlation. After Bonferroni correction, *p* < 0.005 was considered significant.

^b^ Inclusion criteria required concentrations > 1.0 mmol/L.

^c^ Lower limit of detection for the assay is 6.9 μg/mL.

### 3.5 Incidence of Hypervitaminosis D, Ionized Hypercalcemia, and Alterations in Serum PTH Over Study Timepoints

Five dogs (18%) developed hypervitaminosis D during the study. Four out of the five dogs developed elevated 25OHD at T3 (*n* = 3 from cholecalciferol group, *n* = 1 from placebo group; *n* = 2 measured with RIA, *n* = 2 measured with CLIA; Table 2). The dog who developed hypervitaminosis D at T1 had an improved concentration of 25OHD at T2 and a normal serum concentration of 25OHD at T3. All dogs who had serum concentrations of 25OHD above the reference interval at T3 had their concentrations normalize by T4. No dog developed ionized hypercalcemia during the study. Four dogs in the placebo group had elevations in serum PTH at baseline, one of which still had 25OHD below the reference interval and elevation of PTH at T4. Seven dogs in the cholecalciferol group had elevated PTH at baseline, and in 5/7, the PTH concentration remained elevated at T4, despite normalization of 25OHD at that timepoint in 4/5 dogs. One of those dogs had newly elevated PTH at T4 despite normal 25OHD and previously normal PTH at earlier timepoints.

### 3.6 Serum 25OH Measurements

The majority (114/134 [85%]) of 25OHD measurements were performed with the RIA assay. Five dogs in the placebo group and six dogs in the cholecalciferol group had at least one 25OHD measurement with the CLIA. The number of 25OHD measurements by the CLIA did not differ between groups (9/63 [14%] in placebo, 11/71 [15%]) in cholecalciferol group; *p* = 0.81. To look for agreement between the RIA and CLIA, a Bland–Altman method analysis was performed on *n* = 20 dogs. The mean bias was −2.9 ± 45.7. The percentages of dogs with 25OHD within and above the RI at T2–T4 are shown in Table 2.

### 3.7 Outcomes

Two dogs were euthanized during the study, one due to refractory PLE (placebo group), and one due to the development of severe aspiration pneumonia (cholecalciferol group). Long‐term outcomes following completion of the study are available for all additional dogs. Two were euthanized directly related to their PLE, one from the placebo group at 5 months postdiagnosis, and the other from the cholecalciferol group at 6 months postdiagnosis. An additional dog was euthanized due to peri‐articular histiocytic sarcoma at 6 months post‐PLE diagnosis. The remainder of the dogs (23/28; 82%) are alive at the time of manuscript preparation (5–19 months poststudy completion; 8–22 months postdiagnosis).

## 4 Discussion

Dogs with PLE treated with cholecalciferol plus standard treatments had higher concentrations of 25OHD after 2 and 4 weeks of oral supplementation; however, no difference in clinical or biochemical variables was observed over the study period in cholecalciferol‐treated dogs compared to dogs receiving placebo plus standard treatments. Serum albumin was positively correlated with serum 25OHD at T0–T3. A correlation was not observed between VDBP and 25OHD at any study timepoint. Five dogs developed elevated concentrations of 25OHD during the study, all of which returned to the normal reference interval at follow‐up study timepoints. No study dog developed hypercalcemia at any study timepoint.

Vitamin D is a fat‐soluble steroid hormone that has a number of critical roles in health [24]. While the main actions of vitamin D are to increase serum calcium and phosphate and maintain skeletal health, vitamin D has also been found to have specific immunological functions. In the intestine, vitamin D is beneficial for various epithelial cells differentiation and proliferation, and the maintenance of the epithelial barrier [24, 25]. Thus, it is possible that decreased concentrations could contribute to the pathogenesis and/or lack of improvement/resolution of small intestinal disease. In fact, experimental studies in mice have shown that vitamin D supplementation can reduce the severity of chemically induced colitis [26]. In humans with IBD, the prevalence of baseline hypovitaminosis D has been reported to range from 35% to 100% in dogs with CD, and 45% to 50% in dogs with UC [27]. Subsequently, several clinical trials have been performed evaluating various vitamin D treatments in dogs with both CD and UC, some of which have shown positive benefits such as improvements in clinical activity index scores, reductions in inflammatory markers [13], and reduced utilizations of healthcare [14]. An additional placebo‐controlled, randomized, clinical trial of 94 dogs with CD found that the clinical relapse rate was lower in dogs treated with cholecalciferol compared to dogs treated with placebo, though the difference was not statistically different (*p* = 0.06) [28]. However, existing evidence has not consistently identified a significant benefit of vitamin D supplementation in humans with IBD. A recent Cochrane systematic review of the literature investigating vitamin D supplementation in humans with IBD found only 22 randomized controlled clinical trials that met their inclusion criteria. Critical analysis of these trials concluded that there might be fewer clinical relapses in dogs with IBD when receiving vitamin D supplementation compared to placebo, but that no conclusions can be drawn on the effect of vitamin D supplementation on clinical response [29]. Similarly, we did not find convincing evidence that cholecalciferol supplementation improved clinical and biochemical variables in dogs with PLE receiving standard therapy. Furthermore, of the dogs in the placebo group, about 50% had serum 25OHD concentrations within the reference interval 4 weeks poststudy initiation, and approximately 80% were normalized at the final study timepoint, suggesting that treatment of the underlying disease process can lead to resolution of hypovitaminosis D. Given these findings, it could be reasonable for clinicians to monitor for improvement in hypovitaminosis D following initiation of standard treatment of PLE, and only to initiate supplementation in dogs who have persistent decreases in serum 25OHD below an acceptable range. It is important to note that based on the placebo‐controlled nature of our study, dogs with severe ionized hypocalcemia (iCa < 1.0 nmol/L) were not eligible for inclusion in this study, and we would advocate for treatment of severe or clinical ionized hypocalcemia based on published guidelines [30].

The cause of decreased serum 25OHD in dogs with PLE is likely multifactorial. Suggested causes included decreased intake, direct loss, dietary fat malabsorption, and systemic and local inflammation [6, 24]. Multiple previous studies have demonstrated negative correlations between markers of systemic and local inflammation and serum 25OHD concentrations in dogs, as well as a positive correlation between serum 25OHD and albumin concentrations [5, 6, 7, 14]. In this study, a moderate positive correlation was observed between serum albumin and 25OHD concentrations at T0–T3, and at T4 a correlation was observed, but significance was absent after correction for multiple comparisons. A correlation between serum CRP was also observed at T0; however, significance was again lost after correction for multiple testing. There was no correlation between serum CRP and 25OHD at T1–T4, suggesting that systemic inflammation can improve in response to specific therapies such as glucocorticoids and dietary treatments, while serum 25OHD concentrations remain low. Of all the variables tested for a correlation with 25OHD, the correlation with albumin was the strongest and persisted over the course of the study. The reason for the correlation could be due to enteric loss of albumin; however, only about 10%–15% of vitamin D circulates bound to albumin [31]. The majority of vitamin D and its metabolites circulate bound to VDBP [31], therefore, VDBP would also be expected to be low if this was the explanation, though it is important to note that VDBP is more abundant in the circulation than 25OHD. In this study and in previous studies of dogs with CIE [6], acute pancreatitis [32], and proteinuric renal disease [33] VDBP has not been found to be correlated with 25OHD. Additionally, in this study and in dogs with acute pancreatitis [32], there was no correlation between serum albumin and VDBP. Therefore, the relationship between serum albumin and 25OHD in dogs with PLE might not be causal, but instead related to the underlying pathophysiology resulting in a decrease in both, such as intestinal lymphatic dysfunction or malabsorption. This finding does contrast with those of Miller et al. [33], which reported a positive correlation between serum albumin and VDBP in dogs with proteinuric renal disease, despite there being no relationship between VDBP and vitamin D metabolites. Thus, there might be a relationship between serum albumin and VDBP under certain conditions, but it appears to be independent of the relationship between VDBP and 25OHD.

Cholecalciferol was chosen as the vitamin D product for this study as it is inexpensive and widely available. However, it is important to note that other preparations of vitamin D could be superior in dogs. The potency of a dietary 25(OH)D_3_ supplement was five times that of vitamin D_3_ in increasing indicators of vitamin D status in purpose‐bred dogs [34]. An additional study found that vitamin D status was more rapidly and efficiently increased in adult dogs by oral supplementation of 25(OH)D_3_ than D_3_ [35]. The dose of cholecalciferol required to improve 25OHD concentrations in dogs appears to be variable. A study using oral vitamin D3 in dogs with atopic dermatitis reported that the dose ranges needed to improve concentrations of cholecalciferol varied from 300 to 1400 IU/kg per day [15]. Another study demonstrated that a dose of 50 IU/kg/day was effective at raising serum 25OHD concentrations in healthy dogs [36]. At our study dose of 400 IU/kg/day, we were considerably exceeding the allowance recommended by the National Research Council [37]. With this dosage, concentrations of 25OHD improved to > 50 nmol/L by T3 in all dogs in the cholecalciferol group; however, concentrations remained below the RI in several dogs. However, because some of the dogs in the study developed elevated concentrations of 25OHD, a lower initial starting dose is recommended, and we would suggest that close monitoring of vitamin D and calcium concentrations always be performed in dogs receiving cholecalciferol. Given the close association of 25OHD concentrations and serum albumin, it would be reasonable to assume that if the albumin concentration has improved, the 25OHD concentration has also improved. It is unclear why serum PTH concentrations remained high or became increased above the reference interval in several dogs with PLE despite normalization of serum 25OHD. A rebound effect of increasing concentrations of PTH is described in humans with previous decreases in serum calcium [38]; however, many of the dogs in this study never had active serum calcium concentrations below the reference interval. A dietary cause could be considered, but all affected dogs were eating commercially available diets; therefore, the cause remains unclear.

A major limitation of this study is that the original assay used for measurement of 25OHD (RIA) reports cross reactivity with inactive vitamin D metabolites, most importantly, with 24,25‐dihydroxyvitamin D. This cross‐reactivity limits the specificity of the assay and could confound the results since dogs might have high circulating concentrations of 24,25‐dihydroxyvitamin D in some cases [35, 39]. Furthermore, the second assay used in the study does not report cross reactivity of 24,25‐dihydroxyvitamin D; therefore, it is unclear if both assays are measuring similar concentrations of inactive metabolites. Because the original assay kit was discontinued 18 months into the study, all remaining kits were expired, and leftover serum samples were not available for all time points for all dogs, it was necessary for us to utilize the CLIA for all remaining measurements, though we note the limitation. Importantly, however, a similar number of measurements on the CLIA were performed on dogs from each of the groups, and the Bland–Altman analysis looking for agreement between the two methods showed that there was no clinically relevant bias between the two assays. Additional limitations of this study include the use of only one vitamin D product; therefore, limiting speculation to only the use of cholecalciferol in this population. Additionally, based on the clinical nature of this study and the heterogeneous and serious nature of PLE, we felt it would be unethical to treat all dogs in the study with the same diet for the length of the study period; therefore, we elected not to control the diet and to allow the use of home‐cooked diets when necessary. When home‐cooked diets were utilized, they were required to be supplemented with a pure vitamin, mineral, and amino acid supplement. It would have been ideal to quantify the amount of vitamin D in the diets fed. Glucocorticoids can decrease active vitamin D in humans; thus, they could have impacted concentrations of 25OHD in these dogs [40]. Concentrations of 24,25‐dihydroxyvitamin D (which could have been measured on the 25OHD RIA) might also be impacted by glucocorticoids due to an effect on 24‐hydroxylase [41]. Finally, based on this study, no conclusions can be drawn on the relationship of vitamin D status to the pathogenesis of PLE in dogs.

In conclusion, while cholecalciferol at 400 IU/kg/day was effective at increasing serum 25OHD concentrations in dogs with PLE, a clinical benefit of supplementation was not observed. Additionally, 25OHD concentrations improved without vitamin D supplementation in many dogs receiving standard treatment for PLE.

## Disclosure

Authors declare no off‐label use of antimicrobials.

## Ethics Statement

Michigan State University Institutional Animal Care and Use Committee ID # PROTO202200302 and Colorado State University Institutional Animal Care and Use Committee ID #4009. Authors declare human ethics approval was not needed.

## Conflicts of Interest

The authors declare no conflicts of interest.

## Supporting information


**Data S1.** Supporting Information.

**Data S2.** Supporting Information.

**Data S3.** Supporting Information.

**Data S4.** Supporting Information.

**Data S5.** Supporting Information.

**Data S6.** Supporting Information.

**Data S7.** Supporting Information.

**Data S8.** Supporting Information.

Table S1.

Table S2.

Table S3.

Table S4.
