# Erratum to “Treatment of Hypovitaminosis D With Cholecalciferol in Dogs With Protein‐Losing Enteropathies: A Randomized, Double‐Blind, Placebo‐Controlled, Clinical Trial”

**DOI:** 10.1111/jvim.70212

**Published:** 2025-08-22

S. A. Jablonski, S. B. Shropshire, V. E. Watson, et al., “Treatment of Hypovitaminosis D With Cholecalciferol in Dogs With Protein‐Losing Enteropathies: A Randomized, Double‐Blind, Placebo‐Controlled, Clinical Trial,” *Journal of Veterinary Internal Medicine* 39, no. 4 (2025): e70147, https://doi.org/10.1111/jvim.70147.

In the above‐mentioned article, in Section 4 Discussion, second paragraph, lines five, six, and seven the word “dogs” should be replaced with “humans.” The sentences should be as follows.

“In humans with IBD, the prevalence of baseline hypovitaminosis D has been reported to range from 35% to 100% in humans with CD, and 45% to 50% in humans with UC [27]. Subsequently, several clinical trials have been performed evaluating various vitamin D treatments in humans with both CD and UC, some of which have shown positive benefits such as improvements in clinical activity index scores, reductions in inflammatory markers [13], and reduced utilizations of healthcare [14]. An additional placebo‐controlled, randomized, clinical trial of 94 humans with CD found that the clinical relapse rate was lower in humans treated with cholecalciferol compared to humans treated with placebo, though the difference was not statistically different (*p* = 0.06) [28].”

We apologize for this error.

