# Supplementary material for: Treatment of Hypovitaminosis D With Cholecalciferol in Dogs With Protein‐Losing Enteropathies: A Randomized, Double‐Blind, Placebo‐Controlled, Clinical Trial
Source: J Vet Intern Med. 2025 Jun 8;39(4):e70147. doi: 10.1111/jvim.70147 (PMC12146210; doi:10.1111/jvim.70147)
Supplement: Supplementary file 1 — Data S1. Supporting Information. [file JVIM-39-e70147-s003.pdf]

SAFETY DATA SHEET

SECTION 1: PRODUCT IDENTIFICATION

**PRODUCT NAME**	**VITAMIN D3 (Powder)**
**PRODUCT CODE**	**2208**
**SUPPLIER**	**MEDISCA Inc.**<br>Tel.: 1.800.932.1039 | Fax.: 1.855.850.5855<br>626 Tom Miller Road, Plattsburgh, NY, 12901<br>**MEDISCA Pharmaceutique Inc.**<br>Tel.: 1.800.665.6334 | Fax.: 514.338.1693<br>4509 Rue Dobrin, St. Laurent, QC, H4R 2L8<br>**MEDISCA Australia PTY LTD**<br>Tel.: 1.300.786.392 | Fax.: 61.2.9700.9047<br>Unit 7, Heritage Business Park<br>5-9 Ricketty Street, Mascot, NSW 2020
**EMERGENCY PHONE**	CHEMTREC Day or Night Within USA and Canada: 1-800-424-9300<br>NSW Poisons Information Centre: 131 126<br>National Chemical Emergency Centre 44(0)1235239670
**RECOMMENDED USES**	Pharmaceutical Manufacturing
**RESTRICTIONS ON USE**	Not applicable

SECTION 2: HAZARDS IDENTIFICATION

**GHS CLASSIFICATION**	Based on available data, the classification criteria are not met.			
**PICTOGRAM**	Not Applicable			
**SIGNAL WORD**	Not applicable			
**HAZARD STATEMENT(S)**	Not applicable			
**ADVERSE PHYSIOCHEMICAL, HUMAN HEALTH AND ENVIRONMENTAL EFFECTS**	Not applicable			
**PRECAUTIONARY STATEMENT(S)**	**Prevention**	Not applicable		
	**Response**	Not applicable		
	**Storage**	Not applicable		
	**Disposal**	Not applicable		
**HMIS CLASSIFICATION**	**Health Hazard**	1	**Flammability**	0
	**Reactivity**	0	**Personal Protection**	B
**NOTES**	May form combustible dust concentrations in air.			

SECTION 3: COMPOSITION/INFORMATION ON INGREDIENTS

CHEMICAL NAME	Not applicable
BOTANICAL NAME	Not applicable
SYNONYM	Not applicable
CHEMICAL FORMULA	$C_{27}H_{44}O$
CHEMICAL FAMILY	Not applicable
CAS NUMBER	Not applicable
ALTERNATE CAS NUMBER	Not applicable
MOLECULAR WEIGHT	384.65

COMPOSITION

CHEMICAL NAME	CAS NUMBER	EC NUMBER	% BY WEIGHT
STARCH SODIUM OCTENYL SUCCINATE	66829-29-6	828-869-9	Not available
VEGETABLE OIL	68956-68-3	273-313-5	Not available
CHOLECALCIFEROL	67-97-0	200-673-2	0.225% - 0.3%
MALTODEXTRIN	9050-36-6	232-940-4	Not available
MIXED TOCOPHEROLS	59-02-9	200-412-2	Not available
SODIUM ASCORBATE	134-03-2	205-126-1	Not available

There are no additional ingredients present which, within the current knowledge of the supplier and in the concentrations applicable, are classified as health hazards and hence require reporting in this section.

NOTES

Chemical characteristics: Vitamin D3 (Cholecalciferol) preparation with an activity of min 100,000 IU/g protected by modified starch and maltodextrin.

Non-hazardous ingredients: 99.7%-99.775%

SECTION 4: FIRST-AID MEASURES

IN CASE OF EYE CONTACT	Flush with copious amounts of water for 15 minutes, separating eyelids with fingers. If irritation persists seek medical aid.
IN CASE OF SKIN CONTACT	Wash with soap & water for 15 minutes. If irritation persists seek medical aid.
IF SWALLOWED	Call a physician. Wash out mouth with water. Do not induce vomiting without medical advice.
IF INHALED	Remove to fresh air. If not breathing, give artificial respiration. If breathing is difficult, give oxygen. Call a physician
MEDICAL ATTENTION AND SPECIAL TREATMENT	Get emergency medical help.
SYMPTOMS CAUSED BY EXPOSURE	Not expected to present a significant hazard under anticipated conditions of normal use.

SECTION 5: FIREFIGHTING MEASURES

SPECIFIC HAZARDS ARISING FROM THE CHEMICAL	Dust explosion hazard
FLAMMABLE PROPERTIES	May be combustible at high temperature
HAZARDOUS COMBUSTION PRODUCTS	Under fire conditions, hazardous fumes will be present.

**SUITABLE & UNSUITABLE EXTINGUISHING MEDIA**	**Small fire:** dry chemical, CO_2 or water spray. **Large fire:** dry chemical, CO_2, alcohol resistant foam or water spray. Do not get water inside containers.
**PROTECTIVE EQUIPMENT AND PRECAUTIONS FOR FIREFIGHTERS**	Wear self-contained breathing apparatus and protective clothing to prevent contact with skin and eyes.

SECTION 6: ACCIDENTAL RELEASE MEASURES

**PERSONAL PRECAUTIONS**	Wear respiratory protection. Avoid dust formation. Avoid breathing vapours, mist or gas. Ensure adequate ventilation. Evacuate personnel to safe areas. Avoid breathing dust.
**METHODS & MATERIAL FOR CONTAINMENT**	On land, sweep or shovel into suitable containers. Minimize generation of dust.
**CLEANUP PROCEDURE**	Do not touch damaged containers or spilled material unless wearing appropriate protective clothing. Wear respirator, chemical safety goggles, rubber boots and heavy rubber gloves. Stop leak if you can do it without risk. Prevent entry into waterways, sewers, basements or confined areas. Shut off all sources of ignition. Evacuate the area. If necessary, employ water fog to disperse the vapors. Absorb the matter with compatible vermiculite or other absorbing material. Place in a suitable container and retain for disposal. Ventilate and clean the affected area. Do not flush into sewerage system or to drains.
**REFERENCE TO OTHER SECTIONS**	See Section 7 for information on safe handling. See Section 8 for information on personal protection equipment. See Section 13 for disposal information.

SECTION 7: HANDLING AND STORAGE

**PRECAUTIONS FOR SAFE HANDLING**	Do not inhale. Avoid contact with eyes, skin and clothing. Avoid prolonged or repeated exposure. Wash thoroughly after handling. Store away from incompatible materials, in a well-ventilated area. Eliminate all sources of ignition. Store in accordance with local regulations. Do not store in unlabeled containers. Containers that have been opened must be carefully resealed and kept upright to prevent leakage. Use appropriate containment to avoid environmental contamination.<br>Take precautionary measures against static discharges.
**CONDITIONS FOR SAFE STORAGE**	Store away from incompatible materials, in a well-ventilated area. Eliminate all sources of ignition. Store in accordance with local regulations. Do not store in unlabeled containers. Containers that have been opened must be carefully resealed and kept upright to prevent leakage. Use appropriate containment to avoid environmental contamination.
**STORAGE CONDITIONS**	Store in original container, tightly sealed, protected from direct sunlight and moisture.

SECTION 8: EXPOSURE CONTROLS/ PERSONAL PROTECTION

Chemical Name: CHOLECALCIFEROL CAS #: 67-97-0

	Country	Limit value-8 hours		Limit value-Short Term		IDLH	REL	Advisory	Notes
		ppm	mg/m³	ppm	mg/m³				
OSHA	USA	N/L	N/L	N/L	N/L	N/L	N/L	N/A	N/A
ACGIH	USA	N/L	N/L	N/L	N/L	N/L	N/L	N/A	N/A
NIOSH	USA	N/L	N/L	N/L	N/L	N/L	N/L	N/A	N/A
WEEL	USA	N/L	N/L	N/L	N/L	N/L	N/L	N/A	N/A
HSIS	Australia	N/L	N/L	N/L	N/L	N/L	N/L	N/A	N/A

	Country	Limit value-8 hours		Limit value-Short Term		IDLH	REL	Advisory	Notes
HSE	UK	N/L	N/L	N/L	N/L	N/L	N/L	N/A	N/A
GESTIS	Add Country	N/L	N/L	N/L	N/L	N/L	N/L	N/A	N/A

Chemical Name: MALTODEXTRIN CAS #: 9050-36-6

	Country	Limit value-8 hours		Limit value-Short Term		IDLH	REL	Advisory	Notes
		ppm	mg/m³	ppm	mg/m³				
OSHA	USA	N/L	N/L	N/L	N/L	N/L	N/L	N/A	N/A
ACGIH	USA	N/L	N/L	N/L	N/L	N/L	N/L	N/A	N/A
NIOSH	USA	N/L	N/L	N/L	N/L	N/L	N/L	N/A	N/A
WEEL	USA	N/L	N/L	N/L	N/L	N/L	N/L	N/A	N/A
HSIS	Australia	N/L	N/L	N/L	N/L	N/L	N/L	N/A	N/A
HSE	UK	N/L	N/L	N/L	N/L	N/L	N/L	N/A	N/A
GESTIS	Add Country	N/L	N/L	N/L	N/L	N/L	N/L	N/A	N/A

Chemical Name: MIXED TOCOPHEROLS CAS #: 59-02-9

	Country	Limit value-8 hours		Limit value-Short Term		IDLH	REL	Advisory	Notes
		ppm	mg/m³	ppm	mg/m³				
OSHA	USA	N/L	N/L	N/L	N/L	N/L	N/L	N/A	N/A
ACGIH	USA	N/L	N/L	N/L	N/L	N/L	N/L	N/A	N/A
NIOSH	USA	N/L	N/L	N/L	N/L	N/L	N/L	N/A	N/A
WEEL	USA	N/L	N/L	N/L	N/L	N/L	N/L	N/A	N/A
HSIS	Australia	N/L	N/L	N/L	N/L	N/L	N/L	N/A	N/A
HSE	UK	N/L	N/L	N/L	N/L	N/L	N/L	N/A	N/A
GESTIS	Add Country	N/L	N/L	N/L	N/L	N/L	N/L	N/A	N/A

Chemical Name: SODIUM ASCORBATE CAS #: 134-03-2

	Country	Limit value-8 hours		Limit value-Short Term		IDLH	REL	Advisory	Notes
		ppm	mg/m³	ppm	mg/m³				
OSHA	USA	N/L	N/L	N/L	N/L	N/L	N/L	N/A	N/A
ACGIH	USA	N/L	N/L	N/L	N/L	N/L	N/L	N/A	N/A
NIOSH	USA	N/L	N/L	N/L	N/L	N/L	N/L	N/A	N/A
WEEL	USA	N/L	N/L	N/L	N/L	N/L	N/L	N/A	N/A
HSIS	Australia	N/L	N/L	N/L	N/L	N/L	N/L	N/A	N/A
HSE	UK	N/L	N/L	N/L	N/L	N/L	N/L	N/A	N/A
GESTIS	Add Country	N/L	N/L	N/L	N/L	N/L	N/L	N/A	N/A

Chemical Name: STARCH SODIUM OCTENYL SUCCINATE CAS #: 66829-29-6

	Country	Limit value-8 hours		Limit value-Short Term		IDLH	REL	Advisory	Notes
		ppm	mg/m³	ppm	mg/m³				
OSHA	USA	N/L	N/L	N/L	N/L	N/L	N/L	N/A	N/A
ACGIH	USA	N/L	N/L	N/L	N/L	N/L	N/L	N/A	N/A
NIOSH	USA	N/L	N/L	N/L	N/L	N/L	N/L	N/A	N/A
WEEL	USA	N/L	N/L	N/L	N/L	N/L	N/L	N/A	N/A
HSIS	Australia	N/L	N/L	N/L	N/L	N/L	N/L	N/A	N/A
HSE	UK	N/L	N/L	N/L	N/L	N/L	N/L	N/A	N/A
GESTIS	Add Country	N/L	N/L	N/L	N/L	N/L	N/L	N/A	N/A

Chemical Name: VEGETABLE OIL CAS #: 68956-68-3

	Country	Limit value-8 hours		Limit value-Short Term		IDLH	REL	Advisory	Notes
		ppm	mg/m³	ppm	mg/m³				
OSHA	USA	N/L	15(1) 5(2)	N/L	N/L	N/L	N/L	N/A	(1) Inhalable fraction (2) Respirable fraction
ACGIH	USA	N/L	N/L	N/L	N/L	N/L	N/L	N/A	N/A
NIOSH	USA	N/L	10(1) 5(2)	N/L	N/L	N/L	N/L	N/A	(1) Total dust (2) Respirable fraction
WEEL	USA	N/L	N/L	N/L	N/L	N/L	N/L	N/A	N/A
HSIS	Australia	N/L	10(1)	N/L	N/L	N/L	N/L	N/A	(1) This value is for inhalable dust containing no asbestos and < 1% crystalline silica.
HSE	UK	N/L	N/L	N/L	N/L	N/L	N/L	N/A	N/A
GESTIS	Belgium	N/L	10	N/L	N/L	N/L	N/L	N/A	N/A
GESTIS	Canada - Ontario	N/L	10	N/L	N/L	N/L	N/L	N/A	N/A
GESTIS	Canada - Quebec	N/L	10	N/L	N/L	N/L	N/L	N/A	N/A
GESTIS	New Zealand	N/L	10(1)	N/L	N/L	N/L	N/L	N/A	(1) The value for inhalable dust containing no asbestos and less than 1% free silica.
GESTIS	Singapore	N/L	10	N/L	N/L	N/L	N/L	N/A	N/A
GESTIS	South Africa Mining	N/L	10	N/L	N/L	N/L	N/L	N/A	N/A
GESTIS	Sweden	N/L	0.2	N/L	N/L	N/L	N/L	N/A	N/A

N/L = Not listed ; N/A = Not Available
PELs are 8-hour TWAs = Limit value - Eight hours
Ceiling or Short-Term TWA = STEL = Limit value - Short term

EXPOSURE GUIDELINES

Consult local authorities for provincial or state exposure limits. Particulates not otherwise regulated, respirable fraction: 5 mg/m³

PERSONAL PROTECTIVE EQUIPMENT	**Eyes:** Wear appropriate protective eyeglasses or chemical safety goggles as described by WHMIS or OSHA's eye and face protection regulations in 29 CFR 1910.133 or European Standard EN166. **Skin:** Wear appropriate gloves to prevent skin exposure. **Clothing:** Wear appropriate protective clothing to minimize contact with skin. **Respirators:** Follow WHMIS or OSHA respirator regulations found in 29 CFR 1910.134 or European Standard EN 149. Use a NIOSH/MSHA or European Standard EN 149 approved respirator if exposure limits are exceeded or if irritation or other symptoms are experienced. **Thermal Hazards:** For products representing a thermal hazard, appropriate Personal Protective Equipment should be used.
SPECIFIC ENGINEERING CONTROLS	Adequate mechanical ventilation. Fumehood, eye wash station, and safety shower.
BIOLOGICAL MONITORING	Not available
CONTROL BANDING	Not available
NOTES	CHOLECALCIFEROL: USP: ELV: TWA: 0.01 mg/m³

SECTION 9: PHYSICAL AND CHEMICAL PROPERTIES

PHYSICAL STATE	Solid
DESCRIPTION	White to light yellow powder, no foreign matter and no odor.
SOLUBILITY	Soluble in water.
ODOR	Odorless
FLAMMABILITY	May be combustible at high temperature

AUTO-IGNITION TEMPERATURE	410 °C, 770 °F	BOILING POINT	Not available	DECOMPOSITION TEMPERATURE	Not available
EVAPORATION RATE	Not available	EXPLOSIVE LIMIT	Not available	FLASH POINT	Not available
log P (OCTANOL-WATER)	Not available	LOWER FLAMMABLE/ EXPLOSIVE LIMIT(S)	Not available	MELTING/FREEZING POINT	Not available
PARTICLE CHARACTERISTICS	Not available	OXIDIZING PROPERTY	Not available	pH	Not available
RELATIVE DENSITY (WATER = 1)	Not available	SPECIFIC GRAVITY	Not available	UPPER FLAMMABLE/ EXPLOSIVE LIMIT(S)	Not available
VAPOR DENSITY (AIR = 1)	Not available	VAPOR PRESSURE	Not available	VISCOSITY	Not available

The physical data presented above are typical values and should not be construed as a specification.

SECTION 10: STABILITY AND REACTIVITY

REACTIVITY	Sensitive to air and light.

CHEMICAL STABILITY	Stable under recommended storage conditions
INCOMPATIBLE MATERIALS	Strong oxidizing agents, Acid chlorides, Acid anhydrides.
HAZARDOUS DECOMPOSITION PRODUCTS	Toxic fumes of carbon monoxide, carbon dioxide, nitrogen oxides and other gases may occur
HAZARDOUS POLYMERIZATION	Will not occur
POSSIBLITY OF HAZARDOUS REACTION	Not established
CONDITIONS TO AVOID	Moisture, sunlight and extreme temperatures.<br>Product is sensitive to air, light and humidity.

SECTION 11: TOXICOLOGICAL INFORMATION

ACUTE TOXICITY	Oral: Rat: LD50: (mg/kg): Not available<br>Dermal: Rabbit LD50: (mg/kg): Not available<br>Inhalation: Rat: LC50: (mg/L/4hr): Not available	
SKIN CORROSION/IRRITATION	Due to lack of data the classification is not possible.	
SERIOUS EYE DAMAGE/EYE IRRITATION	Due to lack of data the classification is not possible.	
RESPIRATORY SENSITIZATION	Due to lack of data the classification is not possible.	
SKIN SENSITIZATION	Due to lack of data the classification is not possible.	
GERM CELL MUTAGENICITY	Due to lack of data the classification is not possible.	
CARCINOGENICITY	OSHA	No component of this product, present at ≥0.1%, is listed.
	NTP	No component of this product, present at ≥0.1%, is listed.
	IARC	No component of this product, present at ≥0.1% has been evaluated.
	California Proposition	No component of this product, present at ≥0.1% is known to the State of California to cause cancer, birth defects, or any other reproductive harm.
ADDITIONAL CARCINOGENICITY INFORMATION	Due to lack of data the classification is not possible.	
REPRODUCTIVE TOXICITY	CHOLECALCIFEROL - May lead to malformations at dose levels that cause maternal toxicity.<br>NOAEL: 0.0095 mg/kg bw/d (Rabbit female, OECD Test Guideline 414)	
SPECIFIC TARGET ORGAN TOXICITY - SINGLE EXPOSURE	Due to lack of data the classification is not possible.	
SPECIFIC TARGET ORGAN TOXICITY - REPEATED EXPOSURE	CHOLECALCIFEROL - NOAEL (Oral, Rat) : 0.06 mg/kg bw/d; Sub-chronic toxicity study (90-day). (OECD Test Guideline 408)	
ASPIRATION HAZARDS	Due to lack of data the classification is not possible.	

**SIGNS AND SYMPTOMS OF EXPOSURE**	ROUTES OF EXPOSURE:<br>Oral, Dermal, Inhalation, Eye contact<br>EARLY ONSET SYMPTOMS RELATED TO EXPOSURE:<br>Not available<br>DELAYED HEALTH EFFECT FROM EXPOSURE:<br>Not available<br><br>CHOLECALCIFEROL:<br>Acute ingestion overdose produces the following symptoms: Nausea, Vomiting, Headache, Weakness, Abdominal pain, Dry mouth, Metallic taste, Loss of appetite, Anorexia, Depression, Dehydration and Muscle Weakness.<br><br>After inhalation of dust: Irritation symptoms in the respiratory tract.<br>After eye contact: Irritation.<br>After the uptake of large quantities: diarrhoea, headache, lack of appetite.

**POTENTIAL HEALTH EFFECTS**	**Inhalation**	May cause respiratory tract irritation.
	**Ingestion**	May be harmful if swallowed.
	**Skin**	May cause skin irritation. Prolonged skin contact may cause skin irritation.
	**Eyes**	May cause eye irritation. Dust contact with the eyes can lead to mechanical irritation.

SECTION 12: ECOLOGICAL INFORMATION

**ECOTOXICITY**	EC50: 48 Hr: Crustacea: (mg/L): Not available<br>LC50: 96 Hr: Fish: (mg/L): Not available<br>EC50: 72 or 96 Hr: Algae (or other aqua plants): (mg/L): Not available
**PERSISTENCE AND DEGRADABILITY**	Not available
**BIOACCUMULATIVE POTENTIAL**	Not available
**MOBILITY IN SOIL**	Not available
**OTHER ADVERSE EFFECTS**	Not available
	This product is not intended to be released into the environment

SECTION 13: DISPOSAL CONSIDERATIONS

**DISPOSAL METHODS**	Dispose of in accordance with federal / local laws and regulations. Avoid release into the environment.

SECTION 14: TRANSPORT INFORMATION

**UN PROPER SHIPPING NAME**	Not dangerous good
**UN NUMBER**	Not applicable
**CLASS**	Not applicable
**PACKING GROUP**	Not applicable

AUSTRALIA

**HAZCHEM**	Not Applicable
**EU**	
**TRANSPORT IN BULK ACCORDING TO ANNEX II OF MARPOL 73/78 AND THE IBC CODE**	Not Listed
**ENVIRONMENTAL HAZARDS**	Not available
**SPECIAL SHIPPING INFORMATION**	Not applicable

SECTION 15: REGULATORY INFORMATION

UNITED STATES REGULATIONS

Chemical Name & CAS	CERCLA 40 CFR Part 302.4	SARA (Title III) 40 CFR	EPA 40 CFR Part 355 Appendix A	EPA 40 CFR Part 355 Appendix B	Right-to-know Pennsylvania	Right-to-know New Jersey	Right-to-know Massachusett	California Prop 65
CHOLECALCIFEROL 67-97-0	N/L	N/L	N/L	N/L	N/L	N/L	N/L	N/L
SODIUM ASCORBATE 134-03-2	N/L	N/L	N/L	N/L	N/L	N/L	N/L	N/L
MIXED TOCOPHEROLS 59-02-9	N/L	N/L	N/L	N/L	N/L	N/L	N/L	N/L
STARCH SODIUM OCTENYL SUCCINATE 66829-29-6	N/L	N/L	N/L	N/L	N/L	N/L	N/L	N/L
VEGETABLE OIL 68956-68-3	N/L	N/L	N/L	N/L	N/L	N/L	N/L	N/L
MALTODEXTRIN 9050-36-6	N/L	N/L	N/L	N/L	N/L	N/L	N/L	N/L

N/L = Not Listed; X = Listed

AUSTRALIAN REGULATIONS

Chemical Name & CAS	Poisons and Therapeutic Goods	Therapeutic Goods Act	Code of Practices - Illicit Drug	Poisons Standard	Work Health and Safety Regulations	Inventory of Industrial Chemicals
CHOLECALCIFEROL 67-97-0	N/L	Listed as Schedule 7	N/L	Listed	N/L	N/L
SODIUM ASCORBATE 134-03-2	N/L	N/L	N/L	N/L	N/L	N/L
MIXED TOCOPHEROLS 59-02-9	N/L	N/L	N/L	N/L	N/L	N/L
STARCH SODIUM OCTENYL SUCCINATE 66829-29-6	N/L	N/L	N/L	N/L	N/L	N/L
VEGETABLE OIL 68956-68-3	N/L	N/L	N/L	N/L	N/L	N/L
MALTODEXTRIN 9050-36-6	N/L	N/L	N/L	N/L	N/L	N/L

N/L = Not Listed

EU REGULATIONS

Chemical Name & CAS	REACH ANNEX XVII	REACH ANNEX XIV	EC 1005/2009	EC 850/2004	EC 1107/2009	PIC - Prior Informed Consent Regulation	EC 2012/18
CHOLECALCIFEROL 67-97-0	N/L	N/L	N/L	N/L	N/L	N/L	N/L
SODIUM ASCORBATE 134-03-2	N/L	N/L	N/L	N/L	N/L	N/L	N/L
MIXED TOCOPHEROLS 59-02-9	N/L	N/L	N/L	N/L	N/L	N/L	N/L
STARCH SODIUM OCTENYL SUCCINATE 66829-29-6	N/L	N/L	N/L	N/L	N/L	N/L	N/L
VEGETABLE OIL 68956-68-3	N/L	N/L	N/L	N/L	N/L	N/L	N/L
MALTODEXTRIN 9050-36-6	N/L	N/L	N/L	N/L	N/L	N/L	N/L

N/L = Not Listed; X = Listed

Any EU regulation not listed above is not applicable to this product.

SUBJECT TO INTERNATIONAL AGREEMENT: Not applicable

SECTION 16: OTHER INFORMATION

REFERENCES: Available upon request

ABBREVIATIONS AND ACRONYMS: **ACGIH** - American Conference of Governmental Industrial Hygienists; **AIHA WEEL** – American Industrial Hygiene Association Workplace Environment Exposure Levels; **CAESAR** – Computer Assisted Evaluation of industrial chemical Substances According to Regulations; **CAS** – Chemical Abstract Service; **CERCLA** – Comprehensive Environmental Response, Compensation, and Liability Act; **EC50** – Effective Concentration, 50%; **EPA** – Environmental Protection Agency; **GHS** – Global Harmonized System; **HMIS** – Hazardous Materials Information System; **HSE** – Health and Safety Executive; **HSIS** – Hazardous Substances Information System; **IARC** – International Agency for Research on Cancer; **IDLH** - Immediately Dangerous to Life or Health; **IRFMN** – Ready Biodegradability Model; **ISS** – Instituto Superiore Sanità; **LC50** – Lethal Concentration, 50%; **LD50** – Lethal Dose, 50%; **MSHA** - Mine Safety and Health Administration; **NIOSH** – National Institute for Occupational Safety and Health; **NTP** – National Toxicology Program; **OSHA PEL** – Occupational Safety & Health Administration Permissible Exposure Limits; **QSAR** – Quantitative Structure-activity relationship; **REL** - Recommended Exposure Limit; **SARA** – Superfund Amendments and Reauthorization Act; **STEL** – Short Term Exposure Limit; **TLV** – Threshold Limit Value; **TWA** – Time Weighted Average; **WHMIS** – Workplace Hazardous Materials Information System

LAST REVISION: 01/2024

SUPERSEDES: 09/2023

For a list of changes to the SDS since the last version, please communicate with MEDISCA at www.medisca.com

DISCLAIMER: This document was created in accordance with OSHA, Safe Work Australia and WHMIS regulations. The above information is believed to be correct but does not purport to be all inclusive and shall be used only as a guide. The information in this document is based on the present state of our knowledge and is applicable to the product with regard to appropriate safety precautions. It does not represent any guarantee of the properties of the product. MEDISCA® shall not be held liable for any damage resulting from handling or from contact with the above product. Recipients of the product must take responsibility for observing existing laws and regulations.

SUPPLEMENTARY INFORMATION

For all country specific requirements not outlined on this Safety Data Sheet, please request Supplementary Page to this Safety Data Sheet.

Medisca®

Medisca
PARTNERS IN WELLNESS

ALLERGEN DECLARATION

Product name: VITAMIN D3 (Powder)
Stock code: 2208
Starting material: Synthetic
Manufactured via: Not applicable

BSE/TSE: Based on the ingredients, the product is in compliance with the note for the guidance on minimizing the risk of transmitting animal spongiform encephalopathy agents via human and veterinary medicinal products.

Vegan: Vitamin D3 crystals, from which the powder is produced, originate from cholesterol acetate, extracted from lanolin found in sheep's wool. It should also be noted that the process to manufacture Vitamin D3 from the cholesterol acetate is synthetic.

GMO: Raw materials are not from GMO source and therefore the finished product is free from genetically modified organisms.

Allergen: Please note that there may be several steps before the raw material is produced and access to data on manufacturing raw materials is not easily obtained for the starting materials and intermediates used in the production process. The following items are not used in the manufacturing process of the product, and although manufacturer does not specifically assay for the presence of the items below, it is unlikely that any traces of these items are present in the starting materials or in the final product.

**The product is unlikely to contain the following -**
☒ Cereals containing gluten and products thereof
☒ Corn and products thereof
☒ Celery and products thereof
☒ Soybeans and products thereof
☒ Yeast
☒ Fish and crustacean shellfish and products thereof
☒ Eggs and products thereof
☒ Dairy Products (Milk, Lactose, Caseinates, Whey)
☒ Peanuts and products thereof
☒ Tree nuts (oils) or derivatives
☒ Mustard and products thereof
☒ Sesame Oil
☒ Preservatives
☒ Sulfites >10ppm
☒ Artificial Colours and Flavours
☒ Beef/Chicken/Pork derivatives

This allergen statement is provided for **informational purposes** only, based on product knowledge and manufacturing process, as instructed to MEDISCA by the manufacturer, and is not meant to be a guarantee of absence of the above stated allergens.

This statement substitutes all previous versions issued for the above-mentioned product(s).
We trust this information, which is made up to the best of our knowledge, will be helpful to you.

With kind regards,
The MEDISCA Team

Version: 04
Issued Date: 11/01/2023
Supersedes Date: 07/09/2021
