# Supplementary material for: Treatment of Hypovitaminosis D With Cholecalciferol in Dogs With Protein‐Losing Enteropathies: A Randomized, Double‐Blind, Placebo‐Controlled, Clinical Trial
Source: J Vet Intern Med. 2025 Jun 8;39(4):e70147. doi: 10.1111/jvim.70147 (PMC12146210; doi:10.1111/jvim.70147)
Supplement: Supplementary file 2 — Data S2. Supporting Information. [file JVIM-39-e70147-s006.pdf]

LetcoMED LET US HELP.

Section 1: Identification

**Common Name/Trade Name**	Microcrystalline Cellulose (AVICEL PH101)	
**Supplier Information**	Letco Medical, LLC<br>1316 Commerce Drive NW<br>Decatur, AL 35601<br>1 (800) 239-5288<br>+1 (734) 843-4693	**IN CASE OF EMERGENCY:**<br>Chemtrec<br>1 (800) 424-9300 **(24 hours)**
**Product Synonym(s)**	Microcrystalline cellulose (INCI Name): MCC, cellulose gel	
**Relevant Use(s) of Product**	Manufacture or Compounding of Substances	

Section 2: Hazards Identification

**Classification of Substance or Mixture**	Not a hazardous substance or mixture.
**Signal Word**	None
**Hazard Statement(s)**	N/A
**Pictogram(s)**	N/A
**Precautionary Statement(s)**	N/A
**Hazards Not Otherwise Classified**	May form combustible dust concentrations in air. Aspiration or inhalation of this product could cause chemical pneumonitis. Excessive inhalation of dust can mechanically impede respiration. Due to the hygroscopic properties of the gums, they can form a paste or gel in the airway.
**Ingredient(s) with Unknown Toxicity**	No data Available

Section 3: Composition/Information on Ingredients

**Chemical Name**	Microcrystalline cellulose (INCI Name): MCC, cellulose gel
**Common Name**	Avicel® PH101 Microcrystalline Cellulose
**CAS Number**	9004-34-6
**Impurities and/or Stabilizing Additives**	No data available

Section 4: First Aid Measures

**General Advice**	No data available
**If Inhaled**	Remove person to fresh air. If breathing is difficult or if discomfort occurs and persists, obtain medical attention.
**In Case of Skin Contact**	Wash off with warm water and soap as a precaution
**In Case of Eye Contact**	Rinse thoroughly with plenty of water, also under the eyelids. Get medical attention if eye irritation develops or persists.
**If Swallowed**	Never give anything by mouth to an unconscious person. Drink plenty of water. Get medical attention if symptoms occur.
**Most Important Symptoms and Effects**	Difficulty breathing. Cough. Aspiration or inhalation of this product could cause chemical pneumonitis. Treatment is symptomatic and supportive.

Section 5: Fire Fighting Measures

**Suitable Extinguishing Media**	Use water spray, alcohol-resistant foam, dry chemical or carbon dioxide.
**Special Hazards Arising From the Substance/Mixture**	Avoid dust formation Fine dust dispersed in air, in sufficient concentrations, and in the presence of an ignition source is a potential dust explosion hazard. Static electricity might be sufficient to ignite dust clouds. Possibility of ignition will depend on the minimum ignition energy (MIE) and the type of operations undertaken with the material. MIE values are not provided in this SDS.
**Special PPE and/or Precautions for Firefighters**	As in any fire, wear self-contained breathing apparatus pressure-demand, MSHA/NIOSH (approved or equivalent) and full protective gear.

Section 6: Accidental Release Measures

**Personal Precautions, Protective Equipment and Emergency Procedures**	Avoid dispersal of dust in the air (i.e., cleaning dust surfaces with compressed air.). Avoid breathing dust. Powder may become slippery when wet. For personal protection see section 8.
**Methods and Materials Used for Containment**	Dust deposits should not be allowed to accumulate on surfaces, as these may form an explosive mixture if they are released into the atmosphere in sufficient concentration. Sweep, vacuum or shovel into suitable containers for disposal. Nonsparking tools should be used. Washdown water is not recommended. Powder may become slippery when wet.
**Cleanup Procedures**	Sweep, vacuum or shovel into suitable containers for disposal. Nonsparking tools should be used. Washdown water is not recommended. Powder may become slippery when wet.

Section 7: Handling and Storage

**Precautions for Safe Handling**	Handle in accordance with good industrial hygiene and safety practice. Minimize dust generation and accumulation. Routine housekeeping should be instituted to ensure that dusts do not accumulate on surfaces. Dry powdered material can build static electricity when subjected to the friction of transfer and mixing operations. Provide adequate precautions, such as electrical grounding and bonding, or inert atmosphere. Ensure adequate ventilation. In case of insufficient ventilation, wear suitable respiratory equipment if release of airborne dust is expected.
**Conditions for Safe Storage**	Store at less than 25 Â°C, in tightly closed containers. Keep out of direct sunlight. Store in dry environment away from heat and sources of ignition, i.e., steam pipes, radiant heaters, hot air vents or welding sparks. Do not store with strong smelling materials.

Section 8: Exposure Controls/Personal Protection

**Components with Workplace Control Parameters**	Microcrystalline cellulose (9004-34-6) ACGIH TLV TWA: 10 mg/m3 OSHA PEL TWA: 15 mg/m3 TWA: 5 mg/m3 NIOSH TWA: 10 mg/m3 TWA: 5 mg/m3 Mexico Mexico: TWA 10 mg/m3 Mexico: STEL 20 mg/m3 Microcrystalline cellulose (9004-34-6) British Columbia TWA: 10 mg/m3 TWA: 3 mg/m3 Quebec TWA: 10 mg/m3 TWA: 5 mg/m3 Ontario TWAEV TWA: 10 mg/m3 Alberta TWA: 10 mg/m3 TWA: 5 mg/m3
**Appropriate Engineering Controls**	It is recommended that all dust control equipment such as local exhaust ventilation and material transport systems involved in the handling of this product contain explosion relief vents or an explosion suppression or an oxygen-deficient environment. Use only appropriately classified electrical equipment and powered industrial trucks. Handle in accordance with good industrial hygiene and safety practice. Protective engineering solutions should be implemented and in use. These recommendations apply to the product as supplied. If the product is used in mixtures, contact an appropriate protective equipment supplier or industrial hygienist for more information.
**PPE - Eye/Face Protection**	Safety glasses.
**PPE - Skin Protection**	Minimize skin contamination by following good industrial hygiene practices. Use gloves if extended exposure is anticipated. Protective gloves
**PPE - Body Protection**	Minimize skin contamination by following good industrial hygiene practices. Use gloves if extended exposure is anticipated. Protective gloves
**PPE - Respiratory Protection**	If exposure limits are exceeded or irritation is experienced, NIOSH/MSHA approved respiratory protection should be worn. Positive-pressure supplied air respirators may be required for high airborne contaminant concentrations. Respiratory protection must be provided in accordance with current local regulations

Section 9: Physical and Chemical Properties

**Appearance**	Dry powder Free flowing powder
**Upper/Lower Flammability or Explosive Limits**	No data available
**Odor**	No data available
**Vapor Pressure**	No data available
**Odor Threshold**	No data available
**Vapor Density**	No data available
**pH**	5.0 - 7.0 (11 % solids dispersion) (in solution)
**Relative Density**	No data available
**Melting Point/Freezing Point**	No data available
**Solubility**	Insoluble in water
**Initial Boiling Point and Boiling Range**	No data available
**Flash Point**	No data available
**Evaporation Rate**	No data available
**Flammability (Solid, Gas)**	No data available
**Partition Coefficient**	No data available
**Auto-Ignition Temperature**	No data available
**Decomposition Temperature**	No data available
**Viscosity**	No data available

Section 10: Stability and Reactivity

**Reactivity**	No data available
**Chemical Stability**	Stable under recommended storage conditions.
**Possibility of Hazardous Reactions**	None under normal processing. Hazardous polymerization does not occur.
**Conditions to Avoid**	Dust formation. Excessive heat. Humid air. Sparks.
**Incompatible Materials**	Oxidizing agents. Strong acids.
**Hazardous Decomposition Products**	Burning produces obnoxious and toxic fumes: Sulfur oxides.

Section 11: Toxicological Information

**Acute Toxicity - LD50 Oral**	LD50 Oral > 5 g/kg (Rat).
**Acute Toxicity - Inhalation**	LC50 Inhalation > 5800 mg/m3 (Rat) 4 h
**Acute Toxicity - Dermal**	LD50 Dermal > 2 g/kg (Rabbit)
**Acute Toxicity - Eye**	No data available
**Skin Corrosion/Irritation**	No data available
**Serious Eye Damage/Irritation**	No data available
**Respiratory or Skin Sensitazation**	No data available
**Germ Cell Mutagenicity**	No known mutagenic or teratogenic effects.
**Carcinogenicity IARC**	Contains no ingredient listed as a carcinogen
**Carcinogenicity ACGIH**	Contains no ingredient listed as a carcinogen
**Carcinogenicity NTP**	Contains no ingredient listed as a carcinogen
**Carcinogenicity OSHA**	Contains no ingredient listed as a carcinogen
**Reproductive Toxicity**	This product does not contain any known or suspected reproductive hazards.
**Specific Target Organ Toxicity - Single Exposure**	None known.
**Specific Target Organ Toxicity - Repeated Exposure**	None noted in chronic animal studies.
**Aspiration Hazard**	Aspiration may cause chemical pneumonitis. Excessive inhalation of dust can mechanically impede respiration.

Section 12: Ecological Information

**Toxicity**	The environmental impact of this product has not been fully investigated.
**Persistence and Degradability**	Expected to biodegrade, based on component information.
**Bio-accumulative Potential**	Bioaccumulation is unlikely
**Mobility in Soil**	No data available
**Other Adverse Effects**	None known.

Section 13: Disposal Considerations

**Waste Treatment Methods Product**	This material, as supplied, is not a hazardous waste according to Federal regulations (40 CFR 261). This material could become a hazardous waste if it is mixed with or otherwise comes in contact with a hazardous waste, if chemical additions are made to this material, or if the material is processed or otherwise altered. Consult 40 CFR 261 to determine whether the altered material is a hazardous waste. Consult the appropriate state, regional, or local regulations for additional requirements.
**Waste Treatment Methods Packaging**	Dispose of in accordance with federal, state and local regulations.
**Special Precautions Landfill or Incinerations**	No data available
**Other Information**	No data available

Section 14: Transport Information

**UN Number**	Not dangerous goods.
**UN Proper Shipping Name**	N/A
**Transport Hazard Class(es)**	N/A
**Packaging Group**	N/A
**Environmental Hazards**	N/A

Section 15: Regulatory Information

U.S. Federal Regulations SARA 313 Section 313 of Title III of the Superfund Amendments and Reauthorization Act of 1986 (SARA). This product does not contain any chemicals which are subject to the reporting requirements of the Act and Title 40 of the Code of Federal Regulations, Part 372 SARA 311/312 Hazard Categories Acute health hazard No Chronic health hazard No Fire hazard No Sudden release of pressure hazard No Reactive Hazard No Clean Water Act This product does not contain any substances regulated as pollutants pursuant to the Clean Water Act (40 CFR 122.21 and 40 CFR 122.42). CERCLA This material, as supplied, does not contain any substances regulated as hazardous substances under the Comprehensive Environmental Response Compensation and Liability Act (CERCLA) (40 CFR 302) or the Superfund Amendments and Reauthorization Act (SARA) (40 CFR 355). There may be specific reporting requirements at the local, regional, or state level pertaining to releases of this material. US State Regulations California Proposition 65 This product does not contain any Proposition 65 chemicals. U.S. State Right-to-Know Regulations Chemical name New Jersey X, Massachusetts, X Pennsylvania, X. Microcrystalline cellulose 9004-34-6 International Inventories Chemical name TSCA (United States) X, DSL (Canada)X, EINECS/ELINC S (Europe)X, ENCS (Japan)X, China (IECSC) X, KECL (Korea) X, PICCS (Philippines) X, AICS (Australia) X, Microcrystalline cellulose 9004-34-6 Mexico - Grade Slight risk, Grade 1 Chemical name Carcinogen Status Mexico Microcrystalline cellulose Mexico: TWA 10 mg/m3 Mexico: STEL 20 mg/m3 NFPA Health Hazards 1 Flammability 1 Instability 0 Special Hazards - HMIS Health Hazards 1 Flammability 1 Physical hazard 0 Personal Protection X

Section 16: Other Information

**Additional Information**	N/A
**Prepared By**	Scarlotte Smith
**Revision Date**	01/24/2019 11:51

Disclaimer

Letco Medical, LLC believes that the above information is correct but does not purport to be all inclusive and shall be used only as a guide. The information in this document is based on the present state of our knowledge and is applicable to the product with regard to appropriate safety precautions. If the product is used as a component in another product, this information may not be applicable. NO WARRANTY OF FITNESS FOR ANY PARTICULAR PURPOSE, WARRANTY OF MERCHANTABILITY OR ANY OTHER WARRANTY, EXPRESSED OR IMPLIED, IS MADE CONCERNING THE INFORMATION PROVIDED ABOVE. Letco Medical shall not be held liable for any loss or damage resulting from handling, storage, use or from contact with the above product.
