# Supplementary material for: Treatment of Hypovitaminosis D With Cholecalciferol in Dogs With Protein‐Losing Enteropathies: A Randomized, Double‐Blind, Placebo‐Controlled, Clinical Trial
Source: J Vet Intern Med. 2025 Jun 8;39(4):e70147. doi: 10.1111/jvim.70147 (PMC12146210; doi:10.1111/jvim.70147)
Supplement: Supplementary file 3 — Data S3. Supporting Information. [file JVIM-39-e70147-s012.pdf]

Vit D vs Placebo Clinical Trial Study Medication Log

Study ID: Rx 318657 - 1 per Day	Received 5-10-2023
Client Name: [redacted]	Pet's Name: [redacted]

Date	Dose Taken?	Time of Dose	Comments	Initials
5-11-23	(Y) N	7:30 Am		SP
5-12-23	(Y) N	7:30 Am		SP
5-13-23	(Y) N	8:30 Am		SP
5-14-23	(Y) N	8:30 Am		SP
5-15-23	(Y) N	7:20 Am		SP
5-16-23	(Y) N	7:30 Am		SP
5-17-23	(Y) N	7:30 Am		SP
5-18-23	(Y) N	7:30 Am		SP
5-19-23	Y (N)		Spending the day @ MSU	
5-19-23	(Y) N		Dr Jablonski gave her VIT D today	
5-20-23	(Y) N	8:00 Am		SP
5-21-23	(Y) N	10:30 Am		SP
5-22-23	(Y) N	7:30 Am		SP
5-23-23	(Y) N	7:45 Am		SP
5-24-23	(Y) N	7:30 Am		SP
5-25-23	(Y) N	7:30 Am		SP
5-26-23	(Y) N	8:30 Am		SP
5-27-23	(Y) N	8:30 Am		SP
5-28-23	(Y) N	8:00 Am	added Heartworm today	HP
5-29-23	(Y) N	8 Am		SP
5-30-23	(Y) N	6:30 Am		SP
5-31-23	(Y) N	7:30 Am		SP

Your Next Appointment is on 5/20/2023. Please bring this document.
