# Supplementary material for: Treatment of Hypovitaminosis D With Cholecalciferol in Dogs With Protein‐Losing Enteropathies: A Randomized, Double‐Blind, Placebo‐Controlled, Clinical Trial
Source: J Vet Intern Med. 2025 Jun 8;39(4):e70147. doi: 10.1111/jvim.70147 (PMC12146210; doi:10.1111/jvim.70147)
Supplement: Supplementary file 4 — Data S4. Supporting Information. [file JVIM-39-e70147-s009.pdf]

Supporting Information F3

IDS 25-Hydroxy Vitamin D RIA for Use in Canine Serum

Performance data

Michigan State University

Vitamin D was measured in canine sera with a commercially available radioimmunoassay (RIA) kit that provides reagents necessary for extraction and quantitation of the analyte. After the addition of extraction reagents, serum proteins and 25-OHD are precipitated, followed by a centrifugation where the supernatant is incubated with I^{125}-labelled 25-OHD and sheep antibody to 25-OHD. The antibody bound tracer is separated from the free by a short incubation with anti-sheep IgG cellulose. The RIA was performed with reagent volumes and incubation times described in the manufacturer's protocol, with slight modification to antibody incubation time. The manufacturer reported 100% cross-reactivity with 25-Hydroxyvitamin D_3, 75% cross-reactivity with 25-Hydroxyvitamin D_2, >100% cross-reactivity with 24,25-Dihydroxyvitamin D_3, <0.01% cross-reactivity with cholecalciferol D_3, and <0.30% cross-reactivity with ergocalciferol (D_2). Per the manufacturer, the analytical sensitivity of the assay, defined as the calculated concentration corresponding to the mean minus 2 standard deviations of 10 replicates of the 0 standard was <3 nmol/L. Aliquots of canine serum samples of 33 and 334 nmol/L were mixed at volume combinations of 9:1, 3:1, 1:1, 1:3, and 1:9 and run as samples in an assay. Recovery rates, expressed as %observed/expected, for the combinations were 100%, 98%, 93%, 100%, and 97% respectively. Aliquots of a canine serum sample containing 279 nmol/L were mixed at a 4:1 rate with solutions containing 0, 25. 64, 160, and 400 nmol/L of vitamin D. The % observed/expected recovery rates of added vitamin D for these mixtures were 92%, 84%, 86%, 95%, and 91% respectively. Assay repeatability was assessed with 4 pools of canine serum with mean concentrations of 56, 131, 260, and 494 nmol/L. The respective intraassay % coefficients of variation (CV) for 10 replicates of these pools were 7%, 5%, 11%, and 10%. The respective interassay %CVs for these pools were 5% (n=7), 5% (n=7), 9% (n=7), and 15% (n=6).
