# Supplementary material for: Treatment of Hypovitaminosis D With Cholecalciferol in Dogs With Protein‐Losing Enteropathies: A Randomized, Double‐Blind, Placebo‐Controlled, Clinical Trial
Source: J Vet Intern Med. 2025 Jun 8;39(4):e70147. doi: 10.1111/jvim.70147 (PMC12146210; doi:10.1111/jvim.70147)
Supplement: Supplementary file 5 — Data S5. Supporting Information. [file JVIM-39-e70147-s001.pdf]

25-Hydroxy Vitamin D CLIA for Use in Canine Serum

Performance data

Michigan State University

25-hydroxyvitamin D concentrations were measured in canine sera using a commercially available direct competitive chemiluminescent immunoassay (DiaSorin Liaison® XL) by the Michigan State University Veterinary Diagnostic Laboratory. The manufacturer reported 100% cross-reactivity with 25OHD Vitamin D_2 and D_3, 1.9% cross-reactivity with Vitamin D_2 and D_3, 6.7% cross-reactivity with 1,25-$(OH)_2$ Vitamin D_2, 9.3% cross-reactivity with 1,25-$(OH)_2$ Vitamin D_3, and 1.3% cross-reactivity with 3-epi-25 OH Vitamin D_3. Intra-assay percent coefficients of variation (%CV) for the low, medium, and high canine pools were 7%, 2%, and 5%. The respective canine inter-assay %CVs were 6%, 4%, and 7%.
