# Supplementary material for: Treatment of Hypovitaminosis D With Cholecalciferol in Dogs With Protein‐Losing Enteropathies: A Randomized, Double‐Blind, Placebo‐Controlled, Clinical Trial
Source: J Vet Intern Med. 2025 Jun 8;39(4):e70147. doi: 10.1111/jvim.70147 (PMC12146210; doi:10.1111/jvim.70147)
Supplement: Supplementary file 6 — Data S6. Supporting Information. [file JVIM-39-e70147-s005.pdf]

IDS-iSYS Intact Parathyroid Hormone kit for Use in Canine Serum

Performance data

Michigan State University

Intact parathyroid hormone, or iPTH, was measured in canine sera with a commercially available automated chemiluminescence kit[a] that utilizes two polyclonal antibodies against human PTH. The first antibody recognizes the C-terminal region, amino acids 39-84, and is used as the capture antibody. Then for detection, the second antibody, an acridinium conjugated antibody, recognizes the N-terminal region, amino acids 13-34. In this assay, both the full length PTH (amino acids 1-84) and the large PTH fragment (amino acids 7-84) will be detected. The manufacturer reported 100% cross-reactivity with PTH (1-84), 60% cross-reactivity with PTH (7-84), and 0.5% cross-reactivity with PTH (1-34). Per the manufacturer, the analytical sensitivity of the assay was determined by utilizing 50 low level samples, which yielded a sensitivity of 0.26 pmol/L. Aliquots of canine serum samples of 1.0 and 34.7 pmol/L were mixed at volume combinations of 9:1, 3:1, 1:1, 1:3, and 1:9 and run as samples in an assay. Recovery rates, expressed as %observed/expected, for the combinations were 103%, 104%, 99%, 100%, and 103% respectively. Assay repeatability was assessed with 3 pools of canine serum with mean concentrations of 1.1, 5.2, and 29.4 pmol/L. The respective intraassay % coefficients of variation (CV) for 10 replicates of these pools were 7%, 3%, and 1%. The respective interassay %CVs for these pools were 16% (n=9), 3% (n=10), and 7% (n=10).

[a] Intact Parathyroid Hormone kit, Immunodiagnostics Systems, Boldon, Tyne & Wear, NE35 9PD, UK
