# Supplementary material for: Treatment of Hypovitaminosis D With Cholecalciferol in Dogs With Protein‐Losing Enteropathies: A Randomized, Double‐Blind, Placebo‐Controlled, Clinical Trial
Source: J Vet Intern Med. 2025 Jun 8;39(4):e70147. doi: 10.1111/jvim.70147 (PMC12146210; doi:10.1111/jvim.70147)
Supplement: Supplementary file 7 — Data S7. Supporting Information. [file JVIM-39-e70147-s010.pdf]

Canine Vitamin D Binding Protein (VDBP) ELISA Kit

Catalog Number: MBS043474

Lot #:08/2024

PRECISION

Intra-assay Precision (Precision within an assay): Three samples of known concentration were tested twenty times on one plate to assess intra-assay precision.

Inter-assay Precision (Precision between assays): Three samples of known concentration were tested in six separate assays to assess inter-assay precision.

	Intra-Assay Precision			Inter-Assay Precision		
Sample	1	2	3	1	2	3
n	20	20	20	6	6	6
Mean (μg/ml)	205.8	303.6	720.0	204.3	303.0	729.3
Standard deviation	16.5	7.86	32.7	13.2	6.36	20.1
CV (%)	8.0	2.6	4.6	6.5	2.1	2.8

RECOVERY

The spike recovery was evaluated by spiking 3 levels of Canine VDBP into five health Rat serum samples. The un-spiked serum was used as blank in these experiments.

The recovery ranged from 75 % to 103 % with an overall mean recovery of 93 %.

LINEARITY

To assess the linearity of the assay, five samples were spiked with high concentration of VDBP in Canine serum and diluted with Sample Diluent to produce samples with values within the dynamic range of the assay.

	Average(%)	Range(%)
1:2	99	89-110
1:4	99	90-109
1:8	98	87-107
1:16	96	86-105

SAMPLE VALUES

Serum– Thirty samples from apparently healthy Canine were evaluated for the presence of VDBP in this assay.

Sample	No.	Range(μg/ml)	Mean(μg/ml)
Serum	30	55.08-268.35	143.61

STANDARD OD VALUE

Standard	Blank	S1	S2	S3	S4	S5	S6
OD	0.056	0.119	0.173	0.267	0.449	0.911	1.876

MyBioSource

Certificate of Analysis

**Catalog No: MBS043474**	
**Product Name:** Canine Vitamin D Binding Protein (VDBP) ELISA Kit	
**Date of Production:** August 26, 2024	**Shelf-Life of Product:** Six Months (2°C-8°C)
**Specification:** 96T	**Quantity:** 8
**Testing Date:** August 27, 2024	**Report Date:** August 27, 2024

**Inspection item**	**Description**	**Result**
Components	(see components table below)	Intact<br>Correct<br>Complete
Label	All Labels	Intact, Correct, Complete
Package	All Packages	Intact, Correct, Complete
Inter-assay CV%:	≤15%	Yes
Intra-assay CV%:	≤15%	Yes
**Conclusion**	Qualified products	

Components:

Items	Materials	48 Tests	96 Tests
1	Plate	48 well plate	96 well plate
2	Standards (6 vial)	0.5ml×6 vials	0.5ml×6 vials
3	Specimen Diluent	3.0ml	6.0ml
4	HRP-Conjugate Reagent	5.0ml	10.0ml
5	20×Wash Solution	15ml	25ml
6	Stop Solution	3.0ml	6.0ml
7	Chromogen Solution A	3.0ml	6.0ml
8	Chromogen Solution B	3.0ml	6.0ml
9	Closure Plate Membrane	2	2
10	Sealed Bags	1	1
11	Instruction	1	1

MyBioSource
