# Supplementary material for: Treatment of Hypovitaminosis D With Cholecalciferol in Dogs With Protein‐Losing Enteropathies: A Randomized, Double‐Blind, Placebo‐Controlled, Clinical Trial
Source: J Vet Intern Med. 2025 Jun 8;39(4):e70147. doi: 10.1111/jvim.70147 (PMC12146210; doi:10.1111/jvim.70147)
Supplement: Supplementary file 8 — Data S8. Supporting Information. [file JVIM-39-e70147-s002.pdf]

Supplementary Information File S8- Diets and additional medications prescribed (medications not mentioned in the text) in dogs with protein-losing enteropathy receiving cholecalciferol vs. placebo for treatment of hypovitaminosis D.

Diets

Cholecalciferol group

- n=6 veterinary therapeutic hydrolyzed diet
- n=4 veterinary therapeutic low fat canned food diet
- n=3 homecooked diets formulated (supplemented with vitamin & minerals)
- n=1 veterinary therapeutic fresh frozen diet
- n=1 veterinary therapeutic combination hydrolyzed, low-fat diet

Placebo

- n=5 veterinary therapeutic hydrolyzed diet
- n=4 veterinary therapeutic low fat canned food diet
- n=2 veterinary therapeutic combination hydrolyzed, low-fat diets
- n= 1 veterinary therapeutic novel/selected protein diet
- n=1 homecooked diet formulated

Medications not mentioned in the text

Cholecalciferol group

- n=1 dog was receiving fluoxetine over duration of study for generalized anxiety
- n=1 omeprazole for 7 days
- n=1 maropitant for 7 days

Placebo group

- n=1 dog was receiving levetiracetam for previously diagnosed idiopathic epilepsy
- n=1 dog was receiving sildenafil and pimobendan for a portion of the study period for treatment of pulmonary hypertension (documented via echocardiogram) theorized to be acute onset associated with pulmonary thromboembolism
