# Supplementary material for: Treatment of Hypovitaminosis D With Cholecalciferol in Dogs With Protein‐Losing Enteropathies: A Randomized, Double‐Blind, Placebo‐Controlled, Clinical Trial
Source: J Vet Intern Med. 2025 Jun 8;39(4):e70147. doi: 10.1111/jvim.70147 (PMC12146210; doi:10.1111/jvim.70147)
Supplement: Supplementary file 9 — Table S1. [file JVIM-39-e70147-s011.pdf]

Supporting Information, Table S1. Breed of dogs with PLE and decreased concentrations of 25-hydroxyvitamin-D (25OHD) treated with cholecalciferol or placebo.

**Cholecalciferol Group**	**n**	**Placebo Group**	**n**
Mixed breed	4	American pitbull	1
Labrador retriever	2	American Staffordshire terrier	1
American pitbull	1	Bernese mountain dog	1
Bernese mountain dog	1	Border collie	1
Chesapeake bay retriever	1	Cavalier King Charles spaniel	1
Goldendoodle	1	English setter	1
Newfoundland	1	Great Pyrenees	1
Jack Russell terrier	1	Jack Russell terrier	1
Papillion	1	Labrador retriever	1
Soft-coated wheaten terrier	1	Miniature pincher	1
Yorkshire terrier	1	Mixed breed	1
		Newfoundland	1
		Rottweiler	1
**Total**	**15**	**Total**	**13**
