# Supplementary material for: Treatment of Hypovitaminosis D With Cholecalciferol in Dogs With Protein‐Losing Enteropathies: A Randomized, Double‐Blind, Placebo‐Controlled, Clinical Trial
Source: J Vet Intern Med. 2025 Jun 8;39(4):e70147. doi: 10.1111/jvim.70147 (PMC12146210; doi:10.1111/jvim.70147)
Supplement: Supplementary file 10 — Table S2. [file JVIM-39-e70147-s004.pdf]

Supporting Information, Table S2 – Baseline (T0) ultrasonographic, and histologic data in dogs with PLE and decreased serum concentrations of 25-hydroxy-vitamin D (25OHD) treated with cholecalciferol or placebo

**AUS findings**	**Cholecalciferol (n=15)**<br>**# of dogs (%) with finding**	**Placebo (n=13)**<br>**# of dogs (%) with finding**
Peritoneal effusion<br>(mild, mod, marked)	9/15 (60%<br>n=6 mild, n=2 mod, n=1 marked	10/13 (77%)<br>n=6 mild, n=2 mod, n=2 marked
Mucosal specking	2/15 (13%)	2/13 (15%)
Hyperechoic mucosal striations	10/15 (67%)	10/13 (77%)
Mucosal thickening	6/15 (40%)	9/13 (69%)

**Histologic findings**	**Cholecalciferol (n=15)**<br>**# of dogs (%) with finding**	**Placebo (n=13)**<br>**# of dogs (%) with finding**
Inflammatory infiltrate<br>(mild, mod, marked)<br>(Type- LP, LPN, LPE)	15/15 (100%)<br>n=2 mild, n=7 mod, n=6 marked<br>n=4 LP, n=10 LPE, n=1 LPN	13/13 (100%)<br>n=0 mild, n=10 mod, n=3 severe<br>n=6 LP, n=6 LPE, n=1 LPN
Lacteal dilation<br>(mild, mod, marked)	15/15 (100%)<br>n= 11 mild, n=2 mod, n=2 marked	12/13 (92%)<br>n=11 mild, n=1 mod
Crypt dilation<br>(mild, mod, marked)	12/15 (80%)<br>n=11 mild, n=1 mod	6/13 (46%)<br>n=6 mild

Abbreviations: AUS, abdominal ultrasound; LP, lymphoplasmacytic; LPE, lymphoplasmacytic with eosinophils; LPN, lymphoplasmacytic with neutrophils; mod= moderate.
