# Supplementary material for: Treatment of Hypovitaminosis D With Cholecalciferol in Dogs With Protein‐Losing Enteropathies: A Randomized, Double‐Blind, Placebo‐Controlled, Clinical Trial
Source: J Vet Intern Med. 2025 Jun 8;39(4):e70147. doi: 10.1111/jvim.70147 (PMC12146210; doi:10.1111/jvim.70147)
Supplement: Supplementary file 11 — Table S3. [file JVIM-39-e70147-s008.pdf]

Supporting Information Table S3. Days between study timepoints in dogs with PLE and decreased concentrations of 25-hydroxyvitamin-D (25OHD) treated with cholecalciferol or placebo.

**Variable**	**Timepoint**	**Cholecalciferol (n=15) Mean ±SD**	**Placebo (n=13) Mean ±SD**	***P*-value[@]**
Days	T0-start study drug	8.5 ± 2.6	8.3 ± 2.7	.87
	T0-T1	22.1 ± 4.1	20.4 ± 2.9	.25
	T1-T2	15.5 ± 2.1	14.9 ± 1.9	.36
	T2-T3	14.8 ± 2.0	16.7 ± 3.1	.08
	T3-T4	43.25 ± 3.7	46.8 ± 7.1	.14

[@]P-value as assessed by *t*-test; SD: standard deviation.
