# Supplementary material for: Treatment of Hypovitaminosis D With Cholecalciferol in Dogs With Protein‐Losing Enteropathies: A Randomized, Double‐Blind, Placebo‐Controlled, Clinical Trial
Source: J Vet Intern Med. 2025 Jun 8;39(4):e70147. doi: 10.1111/jvim.70147 (PMC12146210; doi:10.1111/jvim.70147)
Supplement: Supplementary file 12 — Table S4. [file JVIM-39-e70147-s007.pdf]

Supporting Information Table S4. Correlation between serum albumin and VDBP at T1 (n=28), T2 (n=27), T3 (n=27) and T4 (n=24) in dogs with PLE and decreased concentrations of 25-hydroxyvitamin-D (25OHD) treated with cholecalciferol or placebo.

Timepoint	Spearman correlation score (rho)	***P*-value@**
T0	-.0470	.81
T1	-.1185	.56
T2	.0981	.63
T3	-.1256	.53
T4	-.3409	.10

@P-value as assessed by Spearman correlation
